# LASSIE: simulating large-scale models of biochemical systems on GPUs

**DOI:** 10.1186/s12859-017-1666-0

**Published:** 2017-05-10

**Authors:** Andrea Tangherloni, Marco S. Nobile, Daniela Besozzi, Giancarlo Mauri, Paolo Cazzaniga

**Affiliations:** 1 Department of Informatics, Systems and Communication, University of Milano-Bicocca, Viale Sarca 336, Milano, 20126 Italy; 2 Department of Human and Social Sciences, University of Bergamo, Piazzale Sant’Agostino 2, Bergamo, 24129 Italy; 3 SYSBIO.IT Centre of Systems Biology, Piazza della Scienza 2, Milano, 20126 Italy

**Keywords:** Graphics Processing Unit, GPU computing, Reaction-based model, Deterministic simulation, Numerical integration method, LSODA, Nvidia CUDA, Fine-grained parallelization, Systems biology, Rule-based model

## Abstract

**Background:**

Mathematical modeling and in silico analysis are widely acknowledged as complementary tools to biological laboratory methods, to achieve a thorough understanding of emergent behaviors of cellular processes in both physiological and perturbed conditions. Though, the simulation of large-scale models—consisting in hundreds or thousands of reactions and molecular species—can rapidly overtake the capabilities of Central Processing Units (CPUs). The purpose of this work is to exploit alternative high-performance computing solutions, such as Graphics Processing Units (GPUs), to allow the investigation of these models at reduced computational costs.

**Results:**

LASSIE is a “black-box” GPU-accelerated deterministic simulator, specifically designed for large-scale models and not requiring any expertise in mathematical modeling, simulation algorithms or GPU programming. Given a reaction-based model of a cellular process, LASSIE automatically generates the corresponding system of Ordinary Differential Equations (ODEs), assuming mass-action kinetics. The numerical solution of the ODEs is obtained by automatically switching between the Runge-Kutta-Fehlberg method in the absence of stiffness, and the Backward Differentiation Formulae of first order in presence of stiffness. The computational performance of LASSIE are assessed using a set of randomly generated synthetic reaction-based models of increasing size, ranging from 64 to 8192 reactions and species, and compared to a CPU-implementation of the LSODA numerical integration algorithm.

**Conclusions:**

LASSIE adopts a novel fine-grained parallelization strategy to distribute on the GPU cores all the calculations required to solve the system of ODEs. By virtue of this implementation, LASSIE achieves up to 92× speed-up with respect to LSODA, therefore reducing the running time from approximately 1 month down to 8 h to simulate models consisting in, for instance, four thousands of reactions and species. Notably, thanks to its smaller memory footprint, LASSIE is able to perform fast simulations of even larger models, whereby the tested CPU-implementation of LSODA failed to reach termination. LASSIE is therefore expected to make an important breakthrough in Systems Biology applications, for the execution of faster and in-depth computational analyses of large-scale models of complex biological systems.

**Electronic supplementary material:**

The online version of this article (doi:10.1186/s12859-017-1666-0) contains supplementary material, which is available to authorized users.

## Background

Systems Biology is a multidisciplinary research field relying on the cross-talk between mathematical, computational and experimental tools to investigate the functioning of complex biological systems, and to predict how they might behave in both physiological and perturbed conditions. To this aim, different computational methods—e.g., parameter estimation, sensitivity analysis or reverse engineering [1, 2]—are usually exploited to define or calibrate the mathematical model that describes the system of interest. These methods require the execution of a large number of simulations, each one generally corresponding to a distinct model structure or parameterization, that is, to a different set of molecular interactions or to different initializations of the species amounts and/or reaction constants. As a result, the computational burden required by these computational analyses can rapidly overtake the capabilities of Central Processing Units (CPUs), therefore limiting in-depth computational investigations to small-scale models consisting in a few tens of reactions and molecular species at most. General-purpose Graphics Processing Units (GPUs) can be exploited to overcome these drawbacks. Indeed, they are parallel multi-core co-processors that are drawing an ever-growing attention by the scientific community, since they give access to tera-scale performances on common workstations (and peta-scale performances on GPU-equipped supercomputers [3]). As such, they can markedly decrease the running times required by traditional CPU-based software, still maintaining low-costs and energetic efficiency. As a matter of fact, in the latter years GPUs have been widely adopted as an alternative approach to classic parallel architectures for the parallelization of computational methods in Systems Biology, Computational Biology and Bioinformatics [4].

In this work we propose LASSIE (LArge-Scale SImulator), a novel GPU-accelerated software designed to simulate large-scale reaction-based models of cellular processes, consisting in hundreds or thousands of reactions and molecular species. An example of killer-application of LASSIE would consist in the simulation of rule-based models according to the so-called indirect methods (see [5, 6] for more information), especially when some proteins are characterized by multiple phosphorylation sites or binding domains, a condition that yields a combinatorial explosion of intermediate chemical complexes and chemical reactions [7]. We designed LASSIE as a general “black-box” tool able to simulate, in principle, any large-scale reaction-based biochemical system based on mass-action kinetics (e.g., the ErbB signaling pathways modeled by Chen et al. [8]), given that the available GPU memory is sufficient to accommodate the necessary data structures. However, considering the difficulty in the manual definition of such massive models, LASSIE may be adopted as an efficient simulation engine for rule-based modeling tools (e.g., BioNetGen [9], PySB [10], Kappa [11]). As a matter of fact, rule-based modeling can generate extremely large-scale systems characterized by very long simulation times: LASSIE may represent an enabling tool to prevent the application of advanced computational investigations of such biological models.

In silico simulations allow to determine the quantitative variation of molecular species amount in time and/or in space, by exploiting either deterministic, stochastic or hybrid algorithms [12–14]. In particular, when the concentrations of molecular species is high and the effect of biological noise can be neglected [15], Ordinary Differential Equations (ODEs) represent the typical modeling approach for cellular processes. Given a model parameterization (i.e., the initial state of the system and the set of kinetic parameters), the temporal dynamics of the system can be simulated by solving the ODEs using some numerical integrator, such as Euler or Runge-Kutta methods [16]. Unfortunately, ODEs can be affected by a well-known phenomenon named stiffness [17], which occurs when the system of biochemical reactions is characterized by two well-separated dynamical modes, determined by fast and slow reactions, respectively [18]. Stiffness can cause the step-size of integration algorithms to reach extremely small values, thus increasing the overall running time. To solve this issue, advanced integration methods like LSODA [19] can be exploited, thanks to their capability of efficiently solving stiff systems. LSODA is able to recognize when a system is stiff and to dynamically select between the most appropriate integration algorithm: the Adams methods [16] in the absence of stiffness, and the Backward Differentiation Formulae (BDF) [20] otherwise. Despite the improvement of efficiency granted by LSODA, the numerical integration of the system of ODEs can become excessively burdensome when the numbers of reactions and molecular species increase. LASSIE overcomes this limitation by distributing over thousands of GPU cores all the calculations required by the numerical integration methods it embeds, therefore paving the way for fast simulations of large-scale and stiff models of cellular processes. One interesting feature of GPUs is that they can have different characteristics, both in terms of resources (e.g., amount of high performance memories, number of cores) and computing power (e.g., clock rate). Kernels’ performances transparently scale on different GPUs, since they automatically leverage the additional resources offered by the latest architectures, a characteristic known as transparent scalability.

**Fig. 1:**
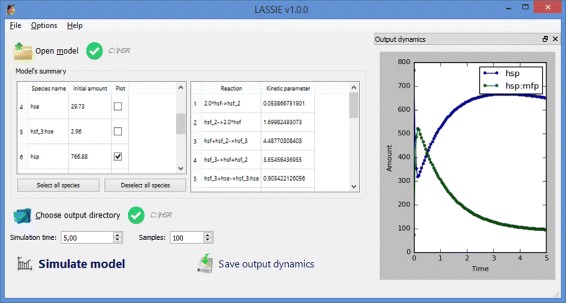
LASSIE’s Graphical User Interface that easily allows the user to (*i*) open a model, (*ii*) visualize its set of species, reactions and parameters, (*iii*) select the output directory, (*iv*) perform a simulation and (*v*) graphically represent the corresponding dynamics

Notably, LASSIE was designed to be a “black-box” deterministic simulator, not requiring any expertise in mathematical modeling nor any GPU programming skill. More precisely, given the formalization of a cellular process as a reaction-based model [21, 22] and assuming mass-action kinetics [23, 24], LASSIE proceeds according to the following workflow: (1) it automatically generates the system of ODEs—one ODE for each molecular species occurring in the biochemical system—according to the biochemical reactions included in the model; (2) it automatically derives the Jacobian matrix, taking advantage of the symbolic derivation, to apply the BDF; (3) it executes the numerical integration of the ODEs by automatically switching between the Runge-Kutta-Fehlberg (RKF) [25] method in the absence of stiffness and first-order BDF (also known as Backward Euler method) [20] in presence of stiffness. We point out that LASSIE is a fully automatic simulator: the user does not need to enter the ODEs directly. On the contrary, the input consists in a set of (parameterized) chemical reactions, specified by means of text files. The corresponding system of ODEs is automatically determined according to the mass-action kinetics, making LASSIE usable without any prior knowledge about ODEs modeling and integration. In order to further simplify the execution of simulations, LASSIE is provided with a user-friendly Graphical User Interface (Fig. 1), whose functioning is described in Additional file 1. A comprehensive description of the input files is provided in Additional file 2.

The computational performances of LASSIE are assessed by measuring the running time required to simulate a set of randomly generated synthetic reaction-based models of increasing size—ranging from 64 to 8192 reactions and species—which is compared to the running time required by a CPU-implementation of LSODA. Moreover, we show the accuracy of LASSIE by comparing its outcome with LSODA outcome for the simulation of a model of the Ras/cAMP/PKA signal transduction pathway in *S. cerevisiae* [26], which is characterized by stiffness.

We highlight that, in general, the implementation of computational methods able to fully exploit the peculiar architecture of GPUs is challenging, since specific programming skills are required and a complete algorithm redesign is often necessary. For instance, the parallelization on the GPU cores can rely either on a coarse-grained or a fine-grained strategy. The first strategy allows to simultaneously run a massive number of independent simulations (each one characterized by, e.g., a different model parameterization); on the contrary, the second strategy consists in the parallelization of all the calculations required by a *single* simulation, an approach that is more suitable for large-scale models. By virtue of the novel *fine-grained* parallelization strategy used to implement LASSIE, our GPU-powered simulator achieves up to 92× speed-up with respect to LSODA.

Coarse-grained parallelizations of deterministic simulations were presented in [27–29]. The simulators proposed in these works allow to reach a speed-up ranging from 28× to 86× with respect to the corresponding CPU-based simulators. Fine-grained parallelizations of stochastic simulations were presented in [30, 31]. Komarov and D’Souza proposed GPU-ODM [30], a fine-grained simulator of large-scale models based on the Stochastic Simulation Algorithm (SSA) [32]. This tool uses special data structures and functionalities to efficiently distribute all calculations over the multiple cores of GPUs. These optimizations allow GPU-ODM to outperform the most advanced CPU-based implementations of SSA. Komarov et al. also proposed a GPU-powered fine-grained implementation of *τ*-leaping [31], an approximate but accurate stochastic algorithm that is, in general, faster than SSA [33]. This tool was shown to be more efficient than its sequential counterpart in the case of extremely large biochemical networks (i.e., characterized by more than 10^5^ reactions). Notably, to the best of our knowledge, no examples of fine-grained deterministic simulators, such as LASSIE, have been proposed so far.

**Fig. 2:**
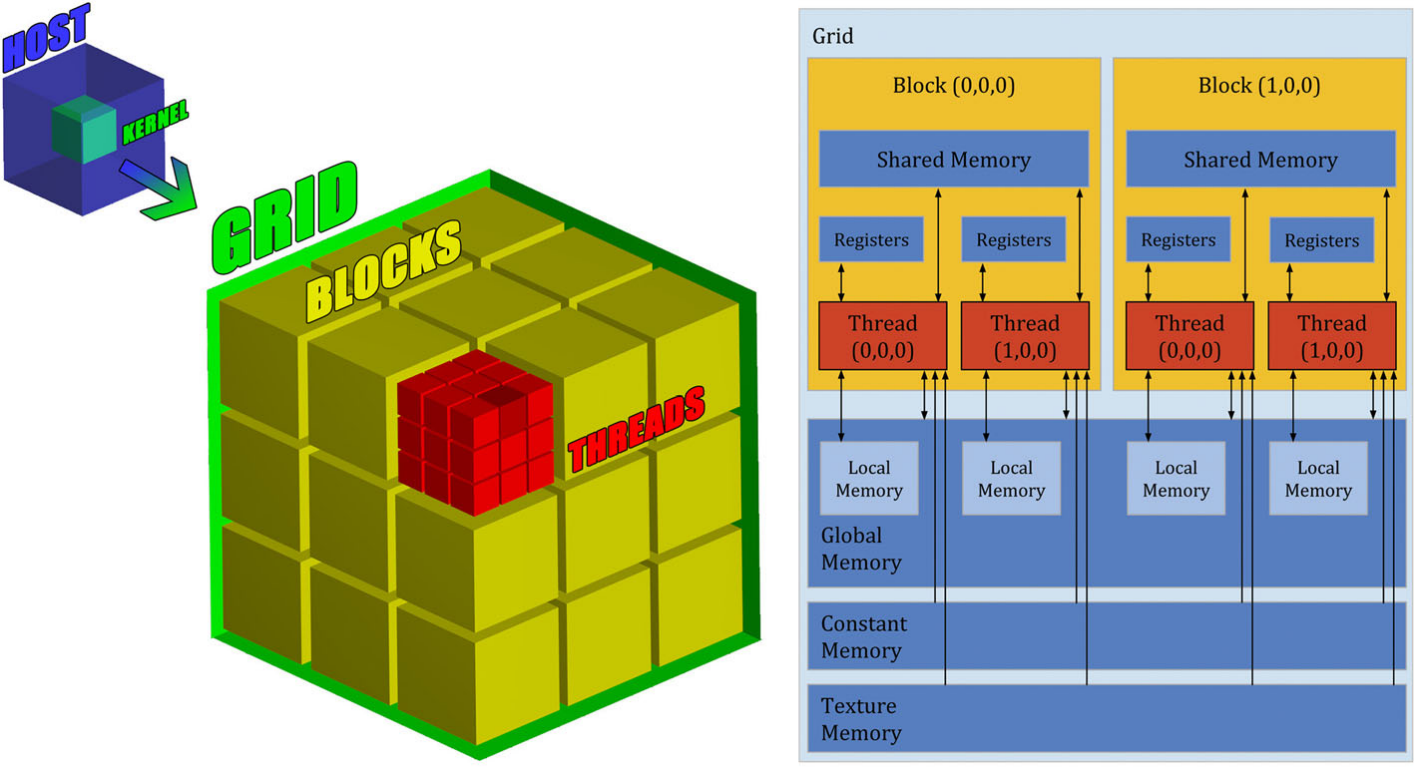
Threads and memory hierarchy of CUDA’s architecture. *Left side*. Thread organization: the host (CPU) launches a single kernel that is executed in multiple threads on the device (GPU). Threads (*red cubes*) are organized in three-dimensional structures called blocks (*yellow cubes*), which belong to three-dimensional grid (*green cube*). The programmer must explicitly define the dimensions of blocks and grids. Whenever a kernel is run by the host, the corresponding grid is created by the device which automatically schedules each block on one free streaming multiprocessor available. This solution allows a transparent scaling of performances on different devices. Moreover, if the machine is equipped with more than one GPU, it is also possible to distribute the workload by launching the kernel on each GPU. *Right side*. Memory hierarchy: in CUDA there are many different memories with different scopes. Each thread has two different kind of private memory: registers and local memories. Threads belonging to the same block can communicate through the shared memory, which has low access latency. The global memory suffers from high access latencies but it is accessible to all threads and it is cached since the introduction of the Fermi architecture. Also the texture and the constant memory are equipped with a cache as well, and all threads can read from these two memories

LASSIE was developed using the most widespread GPU computing library, namely, Nvidia Compute Unified Device Architecture (CUDA). CUDA allows programmers to exploit the GPUs for general-purpose computational tasks (GPGPU computing). Nevertheless, the direct porting of an application to the GPU is usually unfeasible, so that the full exploitation of the computational power and of the massive parallelism of GPUs still represent the main challenges of GPGPU computing. To exploit the CUDA architecture, the programmer implements C/C++ functions (called kernels), which are loaded from the CPU (the host) to one or more GPUs (the devices), and replicated in many copies named threads. CUDA organizes threads in three-dimensional structures called blocks, which belong to three-dimensional structures named grids, as shown in Fig. 2 (left side). CUDA combines the Single Instruction Multiple Data (SIMD) architecture and a flexible multi-threading in order to handle any conditional divergence between threads. Figure 2 (right side) also shows a schematic representation of CUDA’s memory hierarchy: the global memory (accessible from all threads), the shared memory (accessible from threads belonging to the same block), the local memory (each thread has its registers and arrays) and the constant memory (cached and read only). The global memory is large (a few GBs) but suffers from high access latencies; this problem, anyway, was mitigated thanks to the use of L1 cache since the introduction of the Fermi architecture. On the contrary, the constant memory is much smaller (i.e, up to 10 KB for each multi-processor) but faster than the global memory, as well as the shared memory (i.e., up to 112 KB for each multi-processor limited to 48 KB for each block); in particular, the latter should be exploited as much as possible in order to obtain the best performances. Though, the size of the shared memory and its scope restrict the possibility to use it, as only threads belonging to the same block can communicate through the shared memory.

Given the peculiar features of CUDA architecture, GPU programmers should be able to optimize both threads partitioning and memory usage, as well as to redesign the algorithm with appropriate kernels, in order to fully leverage the computational power of these multi-core devices. For instance, in the implementation of LASSIE, the shared memory is not used because several blocks are exploited to solve the system of ODEs, and threads do not communicate data with each other. Moreover, the data structures employed by LASSIE are larger than the total size of the shared memory, thus preventing the possibility to exploit it. In what follows, we show how GPU programming and CUDA features—including built-in support for vector types, which extend the standard C data types to vector—have been exploited to optimize the execution workflow of LASSIE.

The paper is structured as follows. In the next section we briefly introduce the formalism of reaction-based models and provide a general description of LASSIE’s implementation. Then, we discuss the computational performance of LASSIE, showing the speed-up it achieves with respect to LSODA for the simulation of reaction-based models of different sizes. We also analyze how the number of reactions and the number of species affect the performances of LASSIE. We conclude the work with some final remarks about CUDA’s architecture and LASSIE, proposing future improvements of the simulator. LASSIE is available on the GITHUB repository https://github.com/aresio/LASSIE.

## Methods

LASSIE is designed to be a “black-box” deterministic simulator, created to be easily used without any GPU programming or ODEs modeling skills. In this section we describe how LASSIE allows to perform deterministic simulations of large-scale biochemical models, distributing all required calculations on the cores of the GPU. It is worth noting that the parallelization strategy exploited by LASSIE represents one of the novelties of this work, and allowed to achieve the remarkable performance results presented in the next sections. In particular, LASSIE has been developed to solve systems of coupled ODEs specified in the form $\frac {d\mathbf {X}}{dt} = f(t, \mathbf {X})$, where **X**≡**X**(*t*) represents the vector of concentration values at time *t* of all chemical species occurring in the system.

### Reaction-based models and ODEs generation

Reaction-based modeling is a mechanistic, quantitative and parametric formalism to describe and simulate networks of biochemical reactions [22], which was exploited to analyze different signal transduction pathways (see, e.g., [34–39]). A reaction-based model is defined by specifying the set of *N* molecular species {*S*_1_,…,*S*_*N*_} and the set of *M* biochemical reactions {*R*_1_,…,*R*_*M*_} which appear in the cellular process under investigation [22]. A generic reaction is described as follows:

1 $$  R_{i}: \sum\limits_{j=1}^{N} a_{ij}S_{j} \xrightarrow{k_{i}} \sum\limits_{j=1}^{N} b_{ij}S_{j}, \text{~ ~} i=1, \ldots, M,  $$

where *a*_*ij*_, $b_{ij} \in \mathbb {N}$ are the stoichiometric coefficients and $k_{i} \in \mathbb {R}^{+}$ is the kinetic constant associated with *R*_*i*_.

The set of reactions {*R*_1_,…,*R*_*M*_} can be written compactly in the matrix-vector form $\mathbf A \mathbf S \xrightarrow {\mathbf K} \mathbf B \mathbf S$, where **S**=[*S*_1_⋯*S*_*N*_]^*T*^ is the *N*-dimensional column vector of molecular species, **K**=[*k*_1_⋯*k*_*M*_]^*T*^ is the *M*-dimensional column vector of kinetic constants, and **A**,**B**∈**N** ^*M*×*N*^ are the so-called stoichiometric matrices whose (non-negative) elements [*A*]_*i*,*j*_ and [*B*]_*i*,*j*_ correspond to the stoichiometric coefficients *a*_*ij*_ and *b*_*ij*_ of the reactants and the products of all reactions, respectively. Since a reaction simultaneously involving more than two reactants has a probability to take place almost equal to zero, here we consider only first and second-order reactions (i.e., at most two reactant molecules of the same or different species can appear in the left hand side of Eq. 1). For this reason, the matrices **A** and **B** are sparse.

Given an arbitrary reaction-based model and assuming the law of mass-action [24, 40], it is possible to derive the corresponding system of coupled ODEs that describes the variation in time of the species concentrations. Specifically, by denoting the concentration of species *S*_*j*_ at time *t* as *X*_*j*_, where *X*_*j*_∈**R** ^≥0^ for *j*=1,…,*N*, the system of coupled ODEs can be obtained as follows:

2 $$  \frac{d\mathbf{X}}{dt} = (\mathbf B - \mathbf A)^{T} [\mathbf{K} \circ \mathbf{X}^{\mathbf A}],  $$

where **X** is the *N*-dimensional vector of concentration values at time *t* (representing the state of the system at time *t*), the symbol ∘ denotes the entry-by-entry matrix multiplication, and **X** ^**A**^ denotes the vector-matrix exponentiation form [40]. Formally, **X** ^**A**^ is a *M*-dimensional vector whose *i*-th component is given by $X_{1}^{Ai1} \cdots X_{N}^{AiN}$, for *i*=1,…,*M*.

We highlight that each ODE appearing in Eq. 2 is a polynomial function, consisting in at least one monomial that is associated with a specific kinetic constant.

### Data structures and CUDA memory usage

Given a reaction-based model as input, LASSIE automatically generates the systems of ODEs according to Eq. 2 and encodes the matrices **A** and **H**=(**B**−**A**)^*T*^ as two arrays of *short4* CUDA vector types, named **V** **A** and **V** **H**, respectively.

**Fig. 3:**
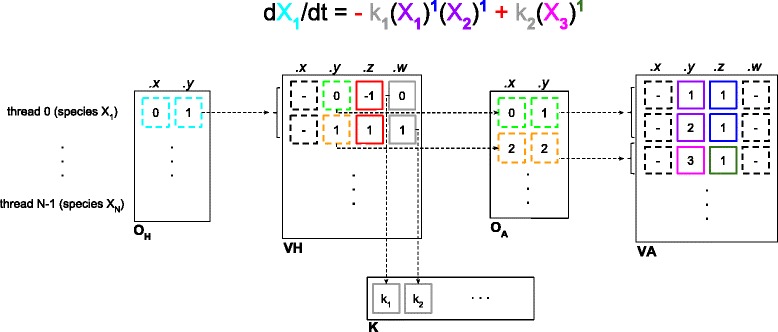
Example of matrix encoding to automatically generate an ODE using LASSIE. All terms of the polynomial function describing the ODE of species *X*_1_ given at the top of the figure are encoded in the components of the data structures *O*_*H*_, **V** **H**, *O*_*A*_, **V** **A** and **K**, as detailed hereby. Notice that only the data structures components with solid borders are used to automatically generate the ODE; the various terms appearing in the ODE are represented with corresponding colors in the data structure components. Matrix encoding starts from matrix *O*_*H*_. Each thread *j*, for *j*=0,…,*N*−1, reads the values stored in the *.x* and *.y* components of *O*_*H*_ (denoted by the *lightblue borders*). In this example, we consider species *X*_1_ that corresponds to thread 0. Each thread fetches the values in **V** **H**, starting from the row indicated by the value stored in the *.x* component of *O*_*H*_, up to the row corresponding to the value stored in the *.y* component. In this example, thread 0 in matrix *O*_*H*_ reads the values contained in the first two rows—i.e., rows 0 and 1—in matrix **V** **H**. Each row of **V** **H** encodes a monomial of an ODE: the *.x* component is not used; the *.y* components (denoted by *green* and *orange borders*) indicate the row numbers of the *O*_*A*_ structure that each thread must read; the *.z* components (*red borders*) indicate the sign and the coefficient of the monomial; the *.w* components (*gray borders*) indicate the positions of the array **K** containing the values of the kinetic constants corresponding to the reactions that the threads are parsing. In this example, the *.z* and *.w* components of **V** **H** allows to derive the coefficients −1*k*_1_ and +1*k*_2_ for the first and the second term of the ODE, respectively. Afterwards, as in the case of *O*_*H*_, each thread fetches the values in **V** **A**, starting from the row indicated by the value stored in the *.x* component of *O*_*A*_, up to the row corresponding to the value stored in the *.y* component of *O*_*A*_. The values stored in the *.y* (*violet* and *fuchsia borders*) and *.z* (*blue* and *dark green borders*) components of **V** **A** correspond to the indexes of the species and the stoichiometric coefficients, respectively, while the *.x* and *.w* components of **V** **A** are left unused. In this example, row 0 in matrix *O*_*A*_ reads the values stored in rows 0 and 1 (*.y* and *.z* components) of matrix **V** **A**, generating the factors (*X*_1_)^1^(*X*_2_)^1^ in the first term of the ODE, while row 1 in matrix *O*_*A*_ reads the values stored in row 2 (*.y* and *.z* components) of matrix **V** **A**, generating the factor (*X*_3_)^1^ in the second term of the ODE. Therefore, in this example, the matrix encoding overall generates the ODE of species *X*_1_ consisting in the sum of two polynomial terms: −*k*_1_(*X*_1_)^1^(*X*_2_)^1^+*k*_2_(*X*_3_)^1^

CUDA vector types are multi-dimensional data ranging from 1 to 4 components, addressed by *.x*, *.y*, *.z*, and *.w*. Since the matrices **A** and **H** are sparse, LASSIE uses compressed data structures created by removing all zero elements from **A** and **H**, in order to save memory and avoid unnecessary readings from the global memory. Namely, let *h*_*ji*_ be the element of **H** at row *j* and column *i*, and *a*_*ij*_ the element of **A** at row *i* and column *j*, for *i*=1,…,*M* and *j*=1,…,*N*. For each non-zero element of **H**, we store into the *.x* and *.y* components of **V** **H** the values *j* and *i*, respectively; the *.z* component of **V** **H** is used to store the element *h*_*ji*_, while the *.w* component stores the index of the kinetic constant associated with that monomial. Similarly, for each non-zero element of **A**, the *.x* and *.y* components of **V** **A** contain the values *i* and *j*, respectively. The value *a*_*ij*_ is stored into the *.z* component of **V** **A**, while the *.w* component is left unused. Note that we exploited the *short4* CUDA vector type rather than the *short3* CUDA vector type, because the former is 8-aligned and requires a single instruction to fetch a whole entry, while the latter is 2-aligned and thus takes three memory operations to read each entry. In order to parse these arrays inside the GPU, we use two additional arrays of *short2* CUDA vector types, named *O*_*H*_ and *O*_*A*_, which store the offsets used to correctly read the entries of the **V** **H** and **V** **A** structures, respectively. The *.x* and *.y* components of each row of *O*_*H*_ contain, respectively, the first index and the last index to access the **V** **H** structure. Each thread uses its own pair of indexes to read the rows of the **V** **H** structure between the first index and the last one. Similarly, *O*_*A*_ stores the indexes that allow to correctly access the **V** **A** structure. Finally, the values of the kinetic constants are stored into an array of type *double*, named **K**. Figure 3 shows an example of the matrix encoding used in LASSIE.

Thanks to these CUDA structures, we obtain a twofold performance improvement: (*i*) at the instruction level, a single instruction is enough to either load or store a multi-word vector. So doing, the total instruction latency for a particular memory transaction is lower and also the bytes per instruction ratio is higher; (*ii*) at the memory controller level, by using vector types a transfer request from a warp has a larger net memory throughput per transaction, yielding a higher bytes per transaction ratio. With a fewer number of transfer requests, the memory controller is able to reduce contentions producing a higher overall memory bandwidth utilization. The only limitation due to *short* data type is that indices are limited to 2^2×8^−1, which means that LASSIE cannot simulate systems larger than 65 536 chemical species and reactions.

### Execution workflow and CUDA kernels

Once that the system of ODEs is generated by reading the input files (see Additional file 2) and appropriately stored according to the CUDA vector types, LASSIE solves it by automatically switching between the Runge-Kutta-Fehlberg (RKF) method [25] in the absence of stiffness, and the Backward Differentiation Formulae (BDF) methods [20] in presence of stiffness. The integration of the systems of ODEs is carried out from an initial time instant *t*_0_, up to a given maximum simulation time *t*_*max*_. In order to reproduce the dynamics of the cellular process described by the ODEs, the concentration values of the molecular species appearing in the reaction-based model are saved at specified time steps within the interval [*t*_0_,*t*_*max*_] (such time steps might correspond, e.g., to the sampling times of laboratory experiments).

**Fig. 4:**
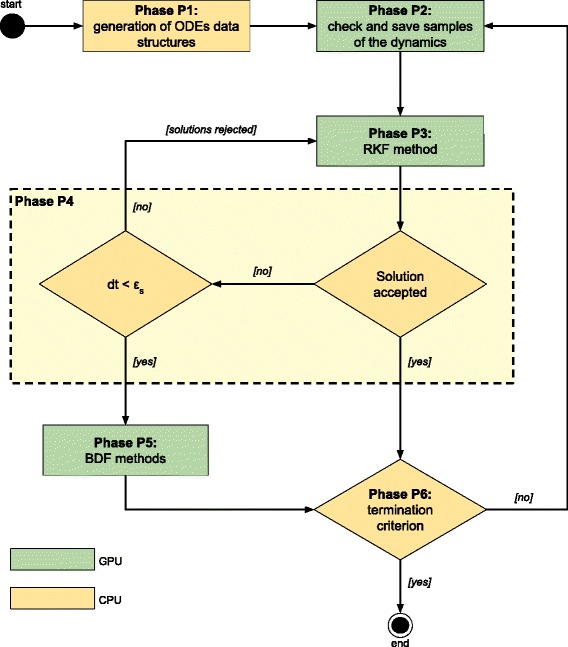
Simplified scheme of LASSIE workflow. The data structures used to encode the system of ODEs are generated in phase *P*_1_. In phase *P*_2_, if the current simulation time *t* corresponds to a specified sampling time instant, then the current concentration values of all molecular species are saved; otherwise, the execution proceeds to the next phase. In phase *P*_3_ each thread derives and solves the corresponding ODE by exploiting the RKF method, while in phase *P*_4_ the RKF solutions are verified: (*i*) if the RKF solutions are rejected, then the integration step-size *dt* is reduced and phase *P*_3_ is executed again; (*ii*) if RKF solutions are rejected but the integration step-size *dt* is too small, then phase *P*_5_ is executed and the system of ODEs is solved using the BDF methods; (*iii*) if the RKF solutions are accepted, the termination criterion is verified during phase *P*_6_ (all phases from *P*_2_ on are iterated until the maximum simulation time *t*_*max*_ is reached)

LASSIE’s workflow consists in 6 distinct phases, as represented in Fig. 4. Note that phases *P*_1_, *P*_4_ and *P*_6_ are executed by the host (yellow boxes in Fig. 4), while *P*_2_, *P*_3_ and *P*_5_ are executed by the device (green boxes in Fig. 4). Overall, phases *P*_2_, *P*_3_ and *P*_5_ rely on 25 different lightweight kernels, which were specifically developed to fully leverage the parallel architecture of the GPU for the implementation of the aforementioned numerical integration methods. We describe hereafter the main design and implementation choices of each phase and their related CUDA kernels, which result in a novel parallelization strategy with respect to state-of-the-art methodologies (see, e.g., [41]).

**Phase** ***P***_**1**_ **.** It implements the generation of all data structures used to encode the ODEs, as described in the previous section. This phase is executed on the host.

**Phase** ***P***_**2**_ **.** It is used to sample and save the system dynamics and it is implemented by means of a single CUDA kernel (**kernel** *K*_1_). In particular, if the current simulation time *t* corresponds to one of the specified sampling time instants, LASSIE saves the concentration values of (possibly, a subset of) all molecular species into an array defined on the GPU. Otherwise, the execution proceeds to the next phase.

**Phase** ***P***_**3**_ **.** It implements the RKF method [42], an explicit integration algorithm with variable step-size used by each thread *j* to solve the *j*-th ODE, for *j*=0,…,*N*−1. This phase is implemented as 9 CUDA kernels.

During this phase, two different approximated states **u**(*t*+*d* *t*) and **w**(*t*+*d* *t*) of the state **X**(*t*+*d* *t*) of the system are generated at each step, thanks to the evaluation of six supplementary values *l*_1_,…,*l*_6_ (see details in Additional file 3). To evaluate the accuracy of **u** and **w** at the current step-size *dt*, LASSIE exploits a user-defined vector tolerance ***ε***∈**R** ^*N*^ (with *ε*_*j*_>0 for all *j*=1,…,*N*), and two additional arrays, **E** **R**,***δ***∈**R** ^*N*^, defined as follows:

3 $$ \begin{aligned} &\mathbf{ER} = \frac{\lvert \mathbf{w}(t+dt) - \mathbf{u}(t+dt) \rvert}{dt}, \; &\boldsymbol{\delta} = 0.84 \left(\frac{\boldsymbol{\varepsilon}}{\mathbf{ER}}\right)^{\frac{1}{4}}. \end{aligned}  $$

If *E* *R*_*j*_≤*ε*_*j*_ for all *j*=1,…,*N*, then **u** is accepted as new state of the system, that is, **X**(*t*+*d* *t*)=**u**(*t*+*d* *t*); otherwise, the solutions **u** and **w** are rejected and recalculated by using a new step-size. The new step-size is computed as *d* *t*=*d* *t*· min{*δ*_1_,…,*δ*_*N*_}, being *δ*_1_,…,*δ*_*N*_ the components of vector ***δ*** (note that the new value of *dt* has to be chosen in order to satisfy the requested error tolerance for all ODEs).

Overall, phase *P*_3_ is implemented by means of the following kernels:

- **kernel** *K*_2_: used to evaluate each ODE at the current state **X** of the system;
- **kernels** *K*_3_ – *K*_8_: each thread *j*, for *j*=0,…,*N*−1, computes the components *l*_1*j*_,…,*l*_6*j*_ of *l*_1_,…,*l*_6_, by invoking **kernel** *K*_2_;
- **kernel** *K*_9_: each thread *j*, for *j*=0,…,*N*−1, computes the components *w*_*j*_ and *u*_*j*_ of the approximated states **u** and **w**, respectively;
- **kernel** *K*_10_: each thread *j*, for *j*=0,…,*N*−1, calculates the components *E* *R*_*j*_ and *δ*_*j*_ of **E** **R** and ***δ***, respectively.

**Phase** ***P***_**4**_ **.** It is used to verify the RKF solutions calculated during phase *P*_3_ and, accordingly, to choose the next phase to be executed: (*i*) if the solutions are rejected and the new step-size *dt* is acceptable (that is, *d* *t*≥*ε*_*s*_, for some *ε*_*s*_>0, e.g., *ε*_*s*_=10^−6^), phase *P*_3_ is executed again exploiting a smaller step-size *dt*; (*ii*) if the solutions are rejected and the new step-size *dt* becomes too small (that is, *d* *t*<*ε*_*s*_), LASSIE executes phase *P*_5_; (*iii*) if all solutions do not violate the specified RKF-tolerance vector ***ε***, then LASSIE executes phase *P*_6_.

Note that point (*ii*) implicitly states that the system of ODEs is considered to be stiff, so that LASSIE automatically switches to phase *P*_5_, where BDF methods are used for the numerical integration. Phase *P*_4_ is executed on the host.

**Phase** ***P***_**5**_ **.** It implements the BDF methods, the most widely used implicit multi-step numerical integration algorithms [43]. LASSIE switches to this phase if and only if the RKF solutions **u** and **w** evaluated during phase *P*_4_ are rejected, and the RKF step-size *dt* becomes smaller than *ε*_*s*_.

The general formula for a BDF can be written as

4 $$ \sum\limits_{i=0}^{q} \alpha_{i} \mathbf{X}(t-t_{i}) = dt \beta_{0} f (t, \mathbf{X}(t)),  $$

where the coefficients *α*_*i*_ (with *α*_0_=1) and *β*_0_ are chosen according to the order *q* of BDF [43], and *dt* is user-defined. Note that, for *q*>6, the absolute stability region of the resulting BDF methods is too small and such BDFs are numerical unstable [44]. Therefore, BDFs with an order *q* greater than 6 are not used. Since each BDF is an implicit method, at each time step it requires the solution of a nonlinear system of equations, which can be solved by using the iterative Newton—Raphson method [45]. This algorithm allows to find successively better approximations *z* of the zeros of a real-valued function *f*(*z*)=0 by using the derivative of *f*(*z*), and it is repeated until a sufficiently accurate value is reached. This idea can be extended to a system of nonlinear equations, by using the Jacobian matrix **J**(*t*,**X**(*t*)) of *f*(*t*,**X**(*t*)), which is the matrix of all first-order partial derivatives. Since the evaluation of the Jacobian matrix at each iteration is computationally expensive, LASSIE actually exploits: (*i*) a modified Newton—Raphson method [46]; (*i* *i*) the LU factorization method [47] (we refer the interested reader to Additional file 3 for technical details). During phase *P*_5_, the Newton-Raphson method is iterated until a user-defined maximum number of iterations *m* *a* *x*_*it*_ is reached, or a sufficiently accurate value is achieved (i.e., smaller than a user-defined tolerance value *ε*_*NR*_).

Overall, phase *P*_5_ is implemented by means of the following kernels:

- **kernel** *K*_11_: each thread *j*, for *j*=0,…,*N*−1, derives the *j*-th row of the Jacobian matrix and evaluates it on the current state of the system **X**;
- **kernel** *K*_12_: the Jacobian matrix is transposed in order to exploit the LU factorization method (accelerated on GPU by the cuBLAS library [48]);
- **kernels** *K*_13_ – *K*_18_: based on the order *q* of the BDF, LASSIE invokes one of these kernels (i.e., **kernel** *K*_13_ for *q*=1, **kernel** *K*_14_ for *q*=2,..., **kernel** *K*_18_ for *q*=6) to calculate the known terms of the linear system;
- **kernels** *K*_19_ – *K*_24_: each **kernel** *K*_(18+*q*)_, *q*=1,…,6, performs the calculations of the *q*-th order BDF;
- **kernel** *K*_25_: it updates the iteration vector needed to execute the Newton-Raphson method (see Additional file 3).

**Phase** ***P***_**6**_ **.** It is used to verify the termination criterion: if the maximum time *t*_*max*_ is reached, then the simulation ends. On the contrary, the execution iterates from phase *P*_2_. This phase is executed on the host.

All the temporary results computed by LASSIE are stored on the GPU, since data transfers between the host and the device are very time consuming. For the same reason, the output data (i.e., the concentration values of molecular species sampled at fixed time instants) are transferred to the host as soon as the whole simulation is completed.

## Results and discussion

In this section we compare the computational performance of LASSIE against LSODA [19], which is generally considered one of the best numerical integration algorithms for deterministic simulations of biological systems, thanks to its capability of dealing with stiff and non-stiff systems. In particular, we exploited the LSODA implementation provided by SciPy library [49] (version 0.15.1), written in C language. LASSIE was run on a machine with a GPU Nvidia GeForce Titan GTX, based on the Kepler architecture and equipped with 2×15 streaming multiprocessors for a total of 5760 cores (clock 837 MHz) and a theoretical peak processing power of 1.3 TFLOPS in double precision. Instead, LSODA was run on GALILEO, a supercomputer created by the Italian consortium CINECA. GALILEO consists of 516 compute nodes, each one equipped with 2 CPUs octa-core Intel Xeon Haswell E5-2630 v3 (clock 2.40 GHz) for a total of 8256 cores, and 128 GB of RAM. Each CPU is capable of about 300 GFLOPS in double precision. In our tests, we exploited one node with 120 GB of RAM distributed over 5 cores.

The computational performance was evaluated by simulating a set of synthetic reaction-based models of increasing size, that is, having a number of reactions and species *M*×*N* arbitrarily chosen in the range from 64×64 to 8192×8192. The models were generated considering the methodology used in [30, 50], which was modified in order to randomly sample the initial concentration of each species with a uniform distribution in the range [0,1), and the kinetic constant of each reaction with a logarithmic distribution in the range [10^−8^,1).

For each model size *M*×*N*, we generated and simulated 30 different synthetic reaction-based models to the aim of measuring the average running time of both LASSIE and LSODA. The simulation of each reaction-based model was performed multiple times, using different settings for the sampling of the time-series. Specifically, in each repetition, we saved either 10,50,100,500 or 1000 samples of the system dynamics of all chemical species, at regular intervals. All simulations were halted at time *t*_*max*_=50 (arbitrary units).

All simulations were executed—independently from the size of the model and the number of samples saved—by setting the following parameters of LASSIE:

- tolerance of RKF method *ε*_*j*_ = 10^−12^, *j*=1,…,*N*;
- first–order BDF method (*q*=1);
- BDF integration step *d* *t*=0.1;
- tolerance of Newton-Raphson method *ε*_*NR*_ = 10^−6^;
- maximum number of iterations allowed during each call of the Newton-Raphson method *m* *a* *x*_*it*_=10^4^;
- initial integration step of RKF method equal to 10^−3^;
- tolerance value to switch between RKF and Backward Euler methods *ε*_*s*_=10^−6^.

The following parameters of LSODA were used to run the simulations:

- relative tolerance equal to 10^−6^;
- absolute tolerance equal to 10^−12^;
- maximum number of internal steps equal to 10^4^.

**Fig. 5:**
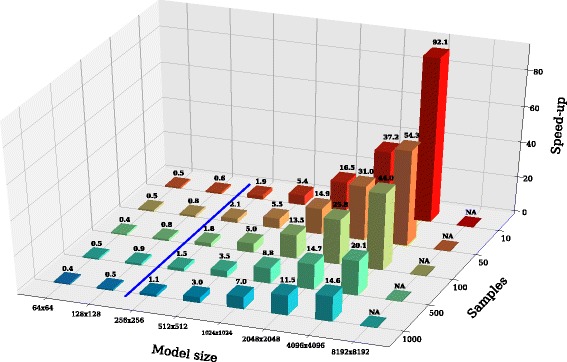
Speed-up values (*z*-axis) achieved by LASSIE with respect to LSODA for the simulation of synthetic models of increasing size, having a number of reactions and of species *M*×*N* (*x*-axis) and characterized by an increasing number of sampling time instants of the system dynamics (*y*-axis). When the value of the speed-up is greater than one, LASSIE is faster than LSODA and vice versa

Table 1 reports the values of the average running times (given in seconds) of LSODA and LASSIE, required for the execution of each set of 30 different synthetic reaction-based models of size *M*×*N*, each time considering 10,50,100,500,1000 samples of the system dynamics of all chemical species. The speed-up values achieved by LASSIE with respect to LSODA are given in Table 1 and graphically represented in Fig. 5, for each tested case; note that when the speed-up value is greater than one, LASSIE is faster than LSODA, and vice versa. The break-even (blue line in Fig. 5) between the performances of LASSIE and LSODA is observed when the number of reactions and chemical species is between 128 and 256. Specifically, in the case of 256×256 model size and 10 samples, the running time of LSODA is almost twice with respect to LASSIE: 1.28 s vs. 0.67 s. In particular, we emphasize that the execution of the simulations for models characterized by 4096 reactions and 4096 species with 10 samples takes, on average, 249.8 s with LSODA and just 2.71 s with LASSIE, resulting in around 92× speed-up. Furthermore, LASSIE allows the simulation of large-scale models (e.g., 8192×8192) thanks to its smaller memory footprint with respect to LSODA, taking just 14.13 s to simulate the model characterized by 8192 reactions and 8192 species with 10 samples. Conversely, the version of LSODA implemented in SciPy library has a high memory footprint that does not allow to simulate models of this size on GALILEO, the supercomputer employed to perform the simulations.

**Table 1:** Average running time (in seconds) of LSODA and LASSIE – and corresponding speed-up value – required for the execution of the set of 30 synthetic reaction-based models of size *M*×*N* (with *M*=*N*), considering 10, 50, 100, 500, 1000 samples of the system dynamics of all chemical species

	10 samples			50 samples			100 samples			500 samples			1000 samples		
*M*×*N*	*LSODA*	*LASSIE*	*Speed-up*	*LSODA*	*LASSIE*	*Speed-up*	*LSODA*	*LASSIE*	*Speed-up*	*LSODA*	*LASSIE*	*Speed-up*	*LSODA*	*LASSIE*	*Speed-up*
64×64	0.257	0.519	0.495	0.288	0.541	0.532	0.220	0.557	0.395	0.307	0.665	0.462	0.303	0.839	0.361
128×128	0.393	0.613	0.641	0.473	0.635	0.745	0.507	0.644	0.787	0.674	0.786	0.858	0.498	0.958	0.520
256×256	1.277	0.669	1.909	1.486	0.696	2.135	1.293	0.727	1.779	1.319	0.905	1.456	1.277	1.122	1.138
512×512	4.313	0.792	5.446	4.629	0.841	5.504	4.559	0.915	4.982	4.300	1.215	3.539	4.669	1.526	3.060
1024×1024	15.753	0.955	16.495	15.707	1.056	14.874	16.201	1.201	13.490	15.982	1.809	8.835	16.647	2.407	6.916
2048×2048	61.824	1.662	37.199	61.748	1.987	31.076	61.762	2.397	25.766	62.307	3.721	14.745	62.742	5.479	11.451
4096×4096	249.839	2.713	92.090*	248.234	4.571	54,306	249.422	5.665	44.029	249.546	12.407	20.113	254.416	17.393	14.627
8192×8192	*NA*	14.134	*NA*	*NA*	26.051	*NA*	*NA*	38.058	*NA*	*NA*	101.91	*NA*	*NA*	129.755	*NA*

^*^Maximum speed-up value

**Fig. 6:**
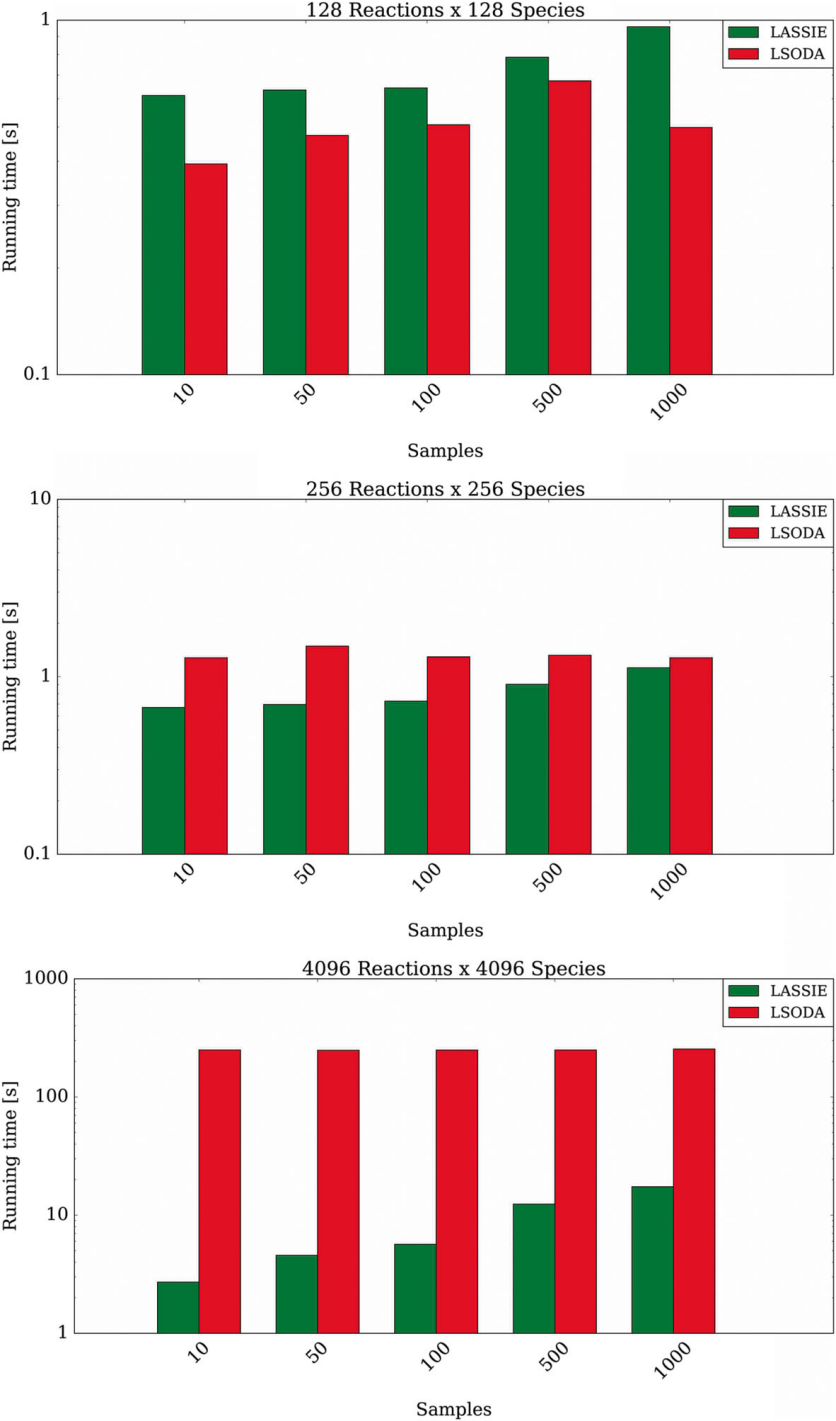
Comparison between the average running time required by LASSIE (*green bars*) and LSODA (*red bars*) to simulate 30 instances of models characterized by 128 reactions and 128 species (*top*), 256 reactions and 256 species (*middle*), 4096 reactions and 4096 species (*bottom*), saving different numbers of sampling time instants of the dynamics. Note that the *y*-axes are in logarithmic scale

Figure 5 also points out how the number of samples of the dynamics affects the performances of LASSIE, due to the different number of accesses to the high-latency global memory. For instance, the speed-up achieved with the model characterized by 4096 reactions and 4096 species decreases to 14.6× with 1000 samples, meaning that the simulations with 1000 samples are around 6× slower than the simulations with 10 samples. In models characterized by 2048 reactions and 2048 species, the speed-up obtained with 10 samples (37.2×) is around 3× larger compared to the one achieved with 1000 samples (11.4×), while in models characterized by 1024 reactions and 1024 species, the speed-up obtained with 10 samples (16.5×) is around 2× larger compared to the one achieved with 1000 samples (6.9×). Finally, Fig. 6 shows that the running time of LASSIE increases with the number of samples, while LSODA is characterized by an almost constant running time, irrespective of the number of samples. It is worth noting that CPU-bound integration methods like LSODA can be more efficient in the case of small-scale models. This is due to two concomitant circumstances. On the one hand, the clock frequency of CPUs is higher than the clock frequency of GPU (2.4 GHz with respect to 837 MHz, in the case of the hardware used to execute our tests). On the other hand, the communication and synchronization between threads can introduce a significant overhead, which is mitigated only when the calculations are distributed over a relevant number of threads; therefore, LASSIE becomes profitable for medium/large-scale models characterized by hundreds of species. Notably, the bigger the model, the greater the speed-up.

As an additional test, we investigated whether the relationship between the number of reactions and the number of species could affect the overall performances of LASSIE. As the number of chemical species corresponds to the number of ODEs, the length of each ODE is roughly proportional to the number of reactions. Since GPUs have a lower clock frequency than CPUs (e.g., in the case of the hardware used for the tests, 837 MHz with respect to 2.4 GHz, respectively), each GPU core is slower than the CPU core to perform a single instruction^1^. For this reason, in order to obtain the highest performances, the calculations on the GPU should be spread across threads as much as possible, while the number of operations performed by each thread should be reduced.

**Fig. 7:**
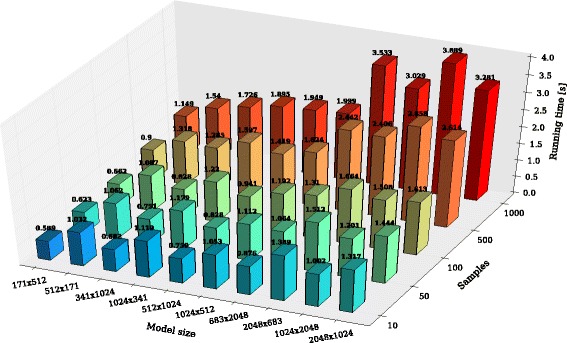
Running time (*z*-axis) of LASSIE for the simulation of synthetic models of increasing size, having a number of reactions and of species *M*×*N* (*x*-axis), with *M*≠*N*, and characterized by an increasing number of sampling time instants of the system dynamics (*y*-axis)

Indeed, as reported in Table 2 and shown in Fig. 7, when the number of chemical species involved in a model is greater than the number of reactions, LASSIE achieves better performances than those obtained in the case of models with a number of chemical species smaller than the number of reactions. For instance, considering the models with *M*×*N* equal to 171×512, the running time of LASSIE is smaller than in the case of the models with size 512×171, irrespective of the number of samples of the system dynamics, thanks to the higher number of threads that are concurrently launched on the GPU in the first case. This is in general valid in all cases with the exception of the models characterized by 2048 chemical species with 500 and 1000 samples of the system dynamics. Here, the average running time of LASSIE is greater than in the case of models with 2048 reactions, since the required number of accesses to the high-latency global memory of the GPU impairs the performances of the simulations.

**Table 2:** Average running time (in seconds) of LASSIE required for the execution of a set of 30 synthetic reaction-based models of size *M*×*N* (with *M*≠*N*), considering 10, 50, 100, 500, 1000 samples of the system dynamics of all chemical species

	10 samples	50 samples	100 samples	500 samples	1000 samples
*M*×*N*	*LASSIE*	*LASSIE*	*LASSIE*	*LASSIE*	*LASSIE*
171×512	0.589	0.623	0.662	0.900	1.149
512×171	1.032	1.062	1.087	1.318	1.540
341×1024	0.682	0.751	0.828	1.285	1.726
1024×341	1.119	1.179	1.220	1.597	1.895
512×1024	0.759	0.828	0.941	1.419	1.949
1024×512	1.053	1.112	1.192	1.624	1.999
683×2048	0.876	1.064	1.310	2.442	3.533
2048×683	1.389	1.512	1.664	2.406	3.029
1024×2048	1.002	1.201	1.508	2.858	3.889
2048×1024	1.317	1.444	1.613	2.614	3.281

**Fig. 8:**
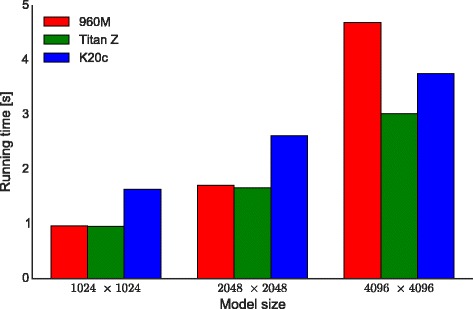
Comparison of the average running times for the simulation of 30 synthetic models characterized by three different sizes, executed with different GPUs: a notebook GPU Nvidia GeForce 960M (*red bars*); a Nvidia GeForce Titan Z (*green bars*); a Tesla-class GPU Nvidia K20c (*blue bars*)

In order to assess the scalability of LASSIE, and of CUDA applications in general, we executed additional tests on different GPUs. Figure 8 shows a comparison of LASSIE’s performance using three different GPU models (Table 3): a notebook video card (Nvidia GeForce 960M, red bars), the Nvidia GeForce Titan Z used throughout the paper (green bars), and a Tesla-class GPU (the Nvidia K20c, blue bars). To compare the speed-up provided by these GPUs we generated 30 different synthetic models (characterized by size *M*×*N* equal to 1024×1024, 2048×2048 and 4096×4096) and calculated the average running time.

**Table 3:** Nvidia GPUs used to assess the scalability of LASSIE

	GeForce GTX 960M	GeForce GTX Titan Z	Tesla K20c
Global memory	4 GB	6 GB	5 GB
Number of streaming multiprocessors	5	15	13
CUDA cores per streaming multiprocessor	128	192	192
Total number of CUDA cores	640	2880	2496
Base clock	1.2 GHz	876 MHz	706 MHz

Our results highlight the importance of two distinct factors on LASSIE’s performances: the GPU’s clock frequency and the amount of available resources (in this case, the cores). As a matter of fact, despite the lower amount of CUDA cores, the GeForce 960M turns out to be competitive on models of moderately large size thanks to its higher clock rate, with respect to the Titan Z and the K20c. When the ODEs largely outnumber the available cores (e.g., for 4096 reactions and chemical species), the GeForce 960M is no longer competitive. This is an example of transparent scalability of CUDA applications: the threads are automatically distributed over the available cores, improving the overall performances, without any user intervention. Moreover, as described in the Background section, threads are organized in blocks that are scheduled on the available multi-processors. Thanks to this characteristic, when the overall number of threads outnumbers the available cores, CUDA automatically creates a queue of blocks that are scheduled on the streaming multi-processors as soon as they become available for computation. Thus, LASSIE can, in principle, simulate any model on any GPU, as long as there is enough memory to store the data structures.

The Tesla K20c is characterized by a large amount of cores that, in the case of 1024×1024 models, are fully exploited only during the simulation of the stiff parts of the dynamics. For the remaining parts of the simulation, half of its cores are actually used for computation with a slower clock rate with respect to the clock rate of the GeForce GPUs. Moreover, Tesla cards exploit Error Correcting Codes (ECC) on memories, ensuring additional checks of correctness to the data against potential corruption from electrical or magnetic interference, at the price of a significant overhead [51]. The ECC was enabled during all tests, partly explaining the reduced performance of the Tesla K20c on very large-scale models with respect to the Titan Z.

We assessed the accuracy of LASSIE by simulating the dynamics of the model of the Ras/cAMP/PKA signaling pathway in yeast presented in [26], and comparing the outcome of LASSIE with the result of the simulation performed with LSODA. We also investigated the influence of LASSIE parameters (e.g., tolerance values) on the running times and quality of the simulated solutions, by exploiting a model representing a chain of isomerizations. The accuracy results—which show an identical dynamics with respect to LSODA using default settings—are presented in the Additional file 4.

As a final remark, we highlight that a fair comparison of GPUs and CPUs is a difficult task, in general, due to their deep architectural differences. The theoretical peak performances of both architectures are difficult to achieve: indeed, developers must implement code to the aim of maximizing the parallelism and the occupancy of the multi-processors, adhering as much as possible to the underlying SIMD computational model in the case of the GPU and exploiting vector instructions in the case of the CPU. However, GPUs allow the temporary divergence of the execution flow of threads, that is, a part of the threads can execute different portions of the code (e.g., the branches of an IF/THEN/ELSE statement). When this situation occurs, some threads get stalled waiting for reconvergence. This mechanism provides the programmer with a certain degree of freedom to abandon the SIMD paradigm, but at the same time it can potentially lead to the complete serialization of the execution affecting the overall performances. Hence, conditional branches should be avoided as much as possible. We also highlight that the usage of registers and shared memory influences the occupancy of the GPU, as these resources are scarce on each streaming multiprocessor. All these circumstances can prevent the achievement of the peak computational power of a GPU.

To this aim, we developed kernels that maximize the parallelism and the occupancy of the multi-processors avoiding threads divergence as much as possible. Moreover, we optimized data structures to store the matrices **A** and **H** that encode the system of ODEs, and CUDA vector types that allow to increase the memory throughput and to reduce the number of memory accesses, all precautions that explain the performance boost achieved with LASSIE.

## Conclusions

In this work we presented LASSIE, a GPU-powered simulator of large-scale biochemical systems based on mass-action kinetics. LASSIE is a “black-box” simulator able to automatically convert reaction-based models of biological systems into the corresponding systems of ODEs. Reaction-based models defined according to the law of mass-action do not hinge upon the use of any approximate kinetics functions (e.g., Michaelis-Menten rate law for enzymatic processes [23], Hill functions for cooperative binding [52], etc.), which are frequently used in Systems Biology for the definition of mathematical models based on differential equations. Although Michaelis-Menten kinetics or Hill functions can be useful in biological modeling, they rely on chemical assumptions that are valid only in certain conditions [53]. Therefore, the reason why we rely on mass-action based models is manifold. On the one hand, since the biological function and biochemical kinetics of all molecular species and all reactions appearing in the model are not approximated nor lumped together in any way, they can be analyzed independently from each other. As a consequence, this allows to determine the influence of every single species and reaction on the overall functioning of the system. On the other hand, the law of mass-action allows to derive a first order ODE for each species appearing in the model: it is worth noting that such ODE is a *polynomial function* that describes how the concentration of that species changes in time, according to all the reactions where it appears either as reactant or product [24]. The presence of polynomial functions simplifies the symbolic derivation that is needed to calculate the Jacobian matrix associated with the ODEs and exploited by the BDF. In addition, as described in the Methods section, polynomials can be efficiently encoded in the memory and parsed GPU-side. As a result, all GPU threads can perform the same task (i.e., polynomial decoding and evaluation), strongly reducing warps’ divergence and the consequent stalling of threads due to serialization, a circumstance that would instead happen if each thread calculated an ODE characterized by an arbitrary kinetics. In order to solve systems of ODEs characterized by stiffness, LASSIE automatically switches between the RKF and the BDF integration methods. LASSIE’s execution flow is partitioned into 25 CUDA kernels, overall distributing the calculations over the available cores in order to fully exploit the massive parallel capabilities of modern GPUs, therefore achieving a relevant reduction of the running time in case of large-scale models.

In order to assess the computational performance of LASSIE, we performed a set of simulation tests using synthetic reaction-based models of increasing size, and we compared LASSIE’s running time with respect to the LSODA numerical integration algorithm implemented in the SciPy library. The break-even between the performances of LASSIE and LSODA was observed when both the numbers of reactions and chemical species is in between 128 and 256. This result indicates that, for biological systems consisting in more than 256 reactions and 256 species, the GPU-powered simulator becomes more convenient than the LSODA algorithm running on CPU. Indeed, in the case of large-scale models, characterized by 4096 reactions and 4096 species, we obtained a considerable 92× speed-up. Moreover, thanks to its smaller memory footprint with respect to LSODA, LASSIE allows the simulation of even larger models, taking just an average of 14.13 s to simulate models characterized by 8192 reactions and 8192 species. On the contrary, LSODA did not allow the simulation of models of this size on the computer we used for the tests, as it crashed because of its very high memory footprint. We also highlight that COPASI [54], one of the most used software in Systems Biology, requires in general longer execution times with respect to the SciPy implementation of LSODA exploited in this work. In addition, COPASI fails when trying to simulate large-scale models. We provide an example of such model—characterized by 4096 reactions and 4096 species—as SBML file in the GITHUB repository.

BDFs are the most used integration algorithms to solve systems of ODEs in case of stiffness. The first–order BDF is a single-step implicit integration method, meaning that the next state of the system depends only on the current state of the system. Higher–order BDFs are multi-step methods, so that the next state of the system relies on multiple previous states of the system (i.e., the number of previous states is equal to the BDF order). This implies that the integration step-size should be the same for all previous states to ensure the correctness of the solution. For this reason, LSODA uses the multi-step Adams methods as explicit methods in addition to BDFs. Conversely, LASSIE uses the RKF method, which is a single-step explicit algorithm with variable step-size, and the Backward Euler method. Other single-step implicit methods belonging to the family of Runge-Kutta methods exist [55], the most known being the families of Lobatto and Radau methods [56]. These methods have been proven to be suitable for stiff systems, thanks to their accuracy and stability [56]. As a future development of LASSIE, we will investigate the feasibility and efficacy of replacing the Backward Euler and, more generally, the BDFs with implicit Runge-Kutta methods [57], in particular Lobatto and Radau methods.

In order to fully exploit the CUDA architecture, the memory hierarchy must be exploited as much as possible. Because of the peculiar sequential structure of both explicit and implicit integration algorithms, LASSIE’s kernel are lightweight and rarely reuse any variables. For this reason, the current implementation only leverages the global memory (characterized by high latencies) and registers to manipulate the mutable data. The shared memory has not been exploited in any way, leaving room for potential future improvements of performances. However, on the GPUs where the L1 cache and the shared memory share the same resources, CUDA allows to express a preference to assign a larger amount of memory to the caching mechanisms. This functionality is enabled by default in LASSIE using the CUDA cudaFuncSetCacheConfig primitive, executed with the cudaFuncCachePreferL1 argument. Also the constant memory has not been used, since all data structures are larger than the total size of this memory. In a future release, we plan to leverage these memories, for instance to store the array of the kinetic constants and the structures containing the offsets used to correctly decode the ODEs. LASSIE currently exploits only a single GPU, even on multi-GPU systems; as a future improvement of this work we plan to extend it in order to support multi-GPU systems, to further increase the size of the models to be simulated.

Finally, an additional goal in the development of LASSIE is to integrate and accelerate the investigation of rule-based models. In the next future, we plan to develop a set of tools that will leverage the functionalities offered by rule-based modeling frameworks [9–11], to convert a rule-based model into a set of reactions. Although rule-based tools already provide internal simulation methods (e.g., PySB allows to perform deterministic simulations using advanced integrators like LSODA), LASSIE can represent a valuable alternative for large-scale models, enabling the investigation of more detailed biological systems, paving the way to potential new discoveries in Systems Biology. LASSIE will be also integrated in COSYS, a free web-based platform for Systems Biology investigation available at http://www.sysbio.it/cosys [58].

## Endnote

^1^ The advances in GPU’s technology will progressively reduce this gap. In middle 2016—with the introduction of the novel Pascal architecture and the 16 nm FinFET manufacturing process—Nvidia presented a GPU with a clock frequency of 1.7 GHz that, theoretically, is expected to double LASSIE’s performances.

## Additional files

Additional file 1 LASSIE Graphical User Interface. (PDF 397 kb)

Additional file 2 LASSIE input files and command line arguments. (PDF 221 kb)

Additional file 3 Implementation of CUDA kernels for LASSIE execution workflow. (PDF 240 kb)

Additional file 4 Simulation accuracy of LASSIE. (PDF 172 kb)

## Abbreviations

BDF: Backward Differentiation Formulae
CPU: Central Processing Unit
CUDA: Compute Unified Device Architecture
ECC: Error Correcting Codes
GPGPU: General-purpose GPU
GPU: Graphics Processing Unit
LASSIE: LArge-Scale SImulator
ODE: Ordinary Differential Equation
RKF: Runge-Kutta-Fehlberg
SIMD: Single Instruction Multiple Data
SSA: Stochastic Simulation Algorithm
