# Supplementary material for: LASSIE: simulating large-scale models of biochemical systems on GPUs
Source: BMC Bioinformatics. 2017 May 10;18:246. doi: 10.1186/s12859-017-1666-0 (PMC5424297; doi:10.1186/s12859-017-1666-0)
Supplement: Supplementary file 1 — LASSIE Graphical User Interface. (PDF 397 kb) [file 12859_2017_1666_MOESM1_ESM.pdf]

ADDITIONAL FILE 1

LASSIE Graphical User Interface

A. Tangherloni, M.S. Nobile, D. Besozzi, G. Mauri, P. Cazzaniga

This document presents the Graphical User Interface (GUI) specifically designed for LASSIE to simplify the execution of simulations. The GUI represents a user-friendly tool that does not require any GPU programming skill or expertise in modeling biological systems with ODEs.

Library dependencies

The following Python libraries are mandatory to use the GUI:

- PyQT4;
- numpy;
- matplotlib.

In addition, to use the beta version of the SBML import module, the python-libsml library is required.

Functioning of the GUI

The GUI provides a visual summary of the information needed by LASSIE (Figure 1):

- the set of chemical species and their initial amounts;
- the set of reactions and their kinetic parameters;
- the total simulation time;
- the set of sampling time instants.

The user needs to select:

1. the input directory containing the model (shortcut: ALT+M);
2. the output directory (shortcut: ALT+C) in which the output dynamics will be saved.

All model information have to be provided using a number of input files, with specific names, placed in the input directory. In particular, the following files are mandatory:

- `left_side` and `right_side`: they specify the stoichiometry of reactants and products, respectively;
- `c_vector`: it contains the kinetic parameters of the reactions;
- `M_0`: it contains the initial amounts of the molecular species.

Further information about the formatting of these and other optional files (e.g., error tolerances) are given in the Additional File 2, along with an example of input files.

Once a model is correctly loaded, and an output directory is selected, their paths will be highlighted in green and the simulation button will be enabled (Figure 2).

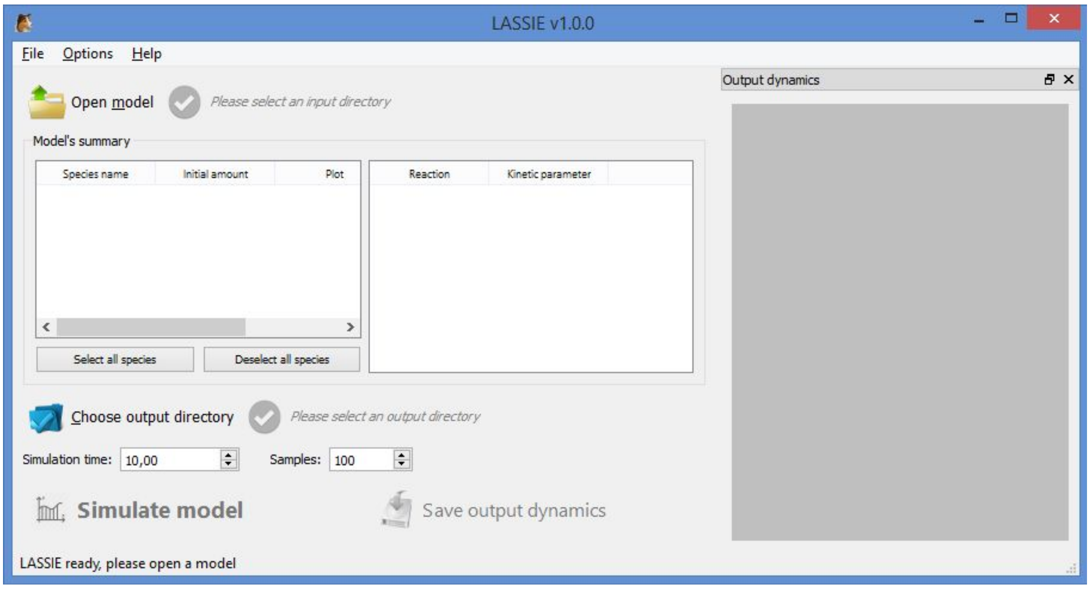

Figure 1: LASSIE's GUI allows the user to open a model by selecting the input directory, and to select the output directory where the simulations outcomes will be saved.

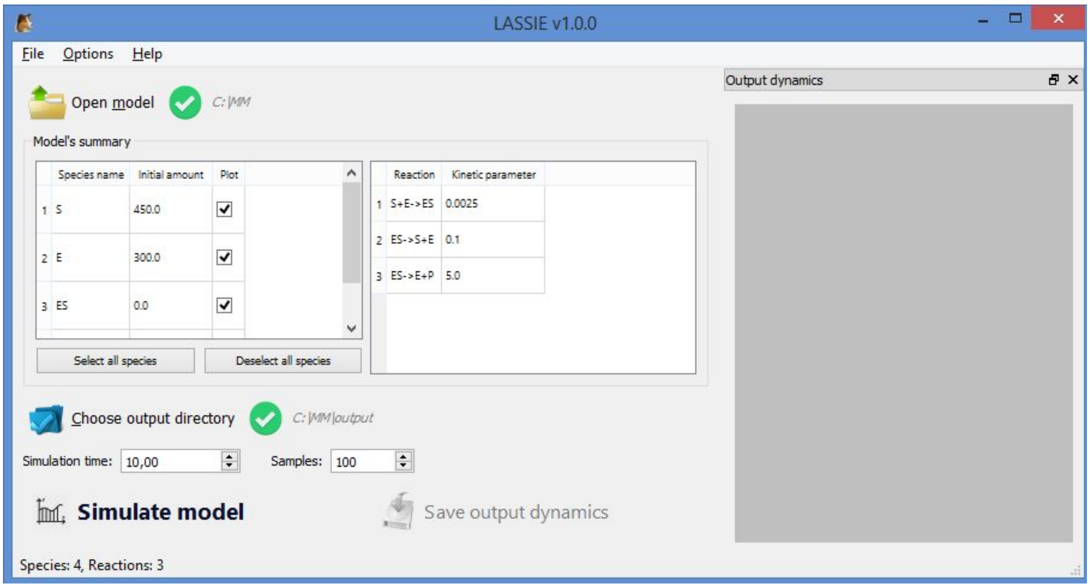

Figure 2: When a model is loaded, its information are summarized in two tables within the "Model's summary": in the left table, the molecular species are listed along with their initial amount; in the right table, the reactions occurring in the model are visualized along with their kinetic constants.

By pressing the "Simulate model" button, LASSIE parses the input files, automatically creates the ODEs (according to the mass-action kinetics law), calculates the Jacobian matrix associated with the ODEs, distributes the integration over the GPU's cores, and collects the output dynamics according to the specified number of sampling time instants. As soon as the simulation ends, the GUI will show the results in the "Output dynamics" detachable widget and the "Save output dynamics" button will be enabled, allowing the user to save the output dynamics to a text file (Figure 3).

The GUI allows to specify the (subset of) species to be plotted, which is a practical feature in the case of large-scale systems characterized by a high number of different molecular species. In order to select the species to plot, the user can click on the corresponding checkboxes in the "Plot" column under the "Model's summary" (Figure 4).

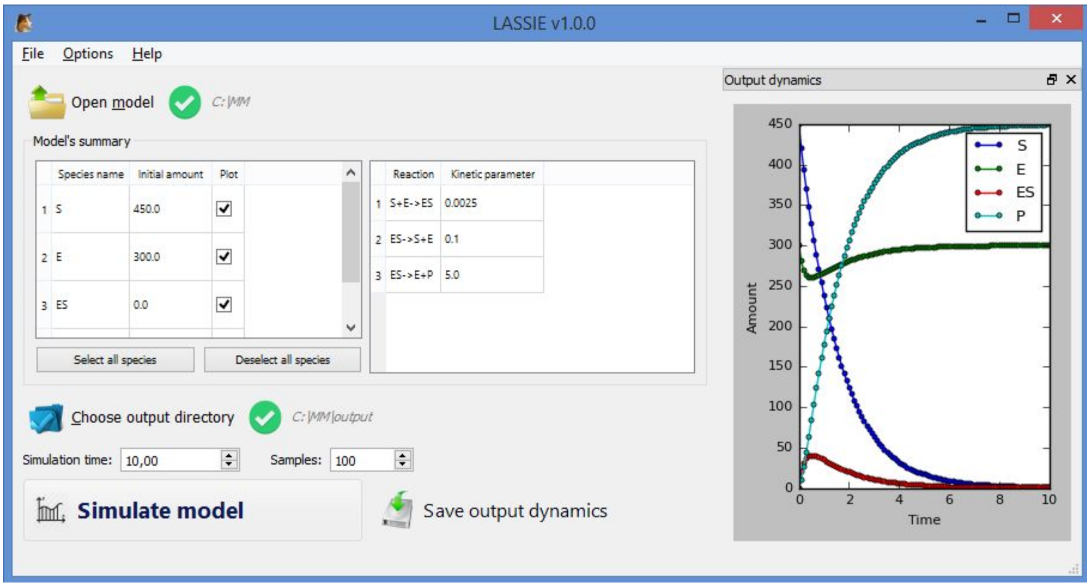

Figure 3: The output dynamics is plotted in a detachable widget (on the right side of the GUI).

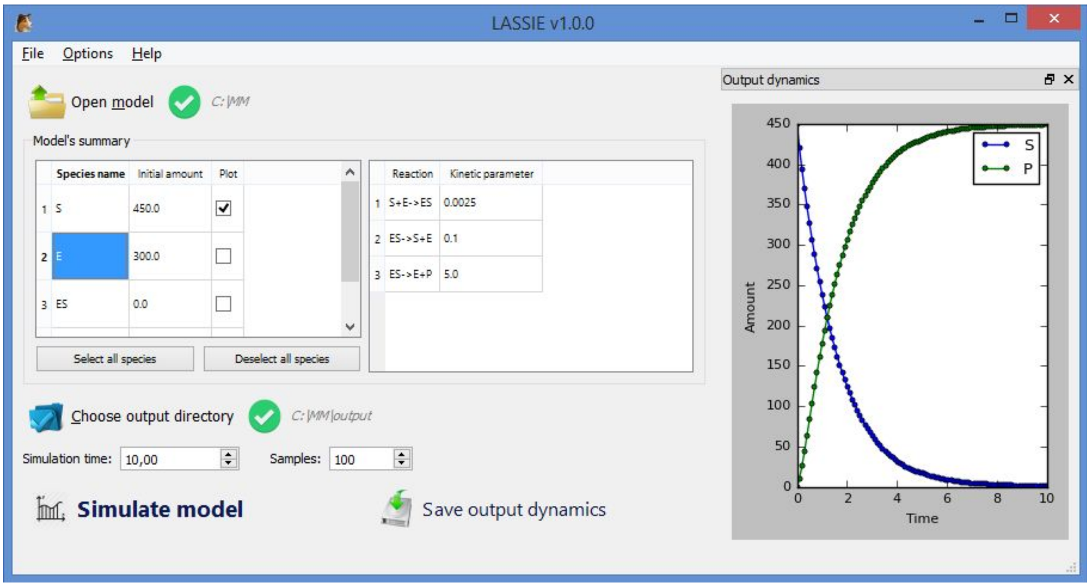

Figure 4: The "Output dynamics" plot can be modified (by adding or removing species) by clicking on the "Plot" checkbox in the molecular species list.

Finally, an information dialog about LASSIE can be opened by clicking the Help > About LASSIE menu (Figure 5).

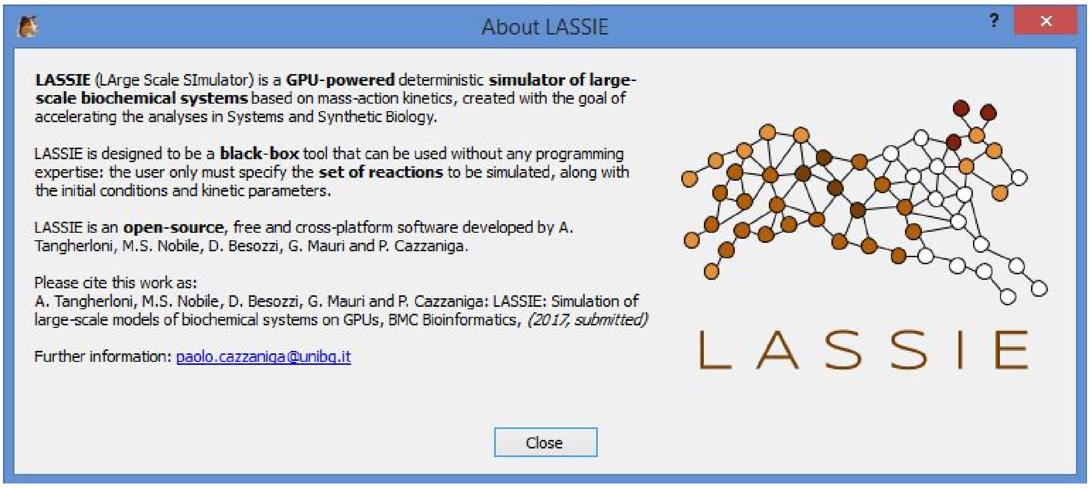

Figure 5: Information dialog about LASSIE.

The GUI represents an alternative way to run simulations with LASSIE, with respect to the command line. At the present time, the GUI supports the following functionalities:

- it provides a visual way to load and simulate a reaction-based model based on mass-action kinetics[1];
- it provides a visual way to specify the total simulation time and the number of sampling time instants of the dynamics to be saved;
- it shows a summary of the main information about the loaded model;
- it plots the output dynamics of the selected species and allows to save the output dynamics to file.

plan to extend the GUI in the next future, by introducing some additional functionalities:

- support for PySB, BioNetGen and Kappa rule-based models (they will be transparently converted into reaction-based models and simulated by LASSIE);
- complete support for de novo model editing;
- support for species, reactions and parameters editing;
- advanced analysis tools (e.g., sensitivity analysis, parameter estimation);
- select and show the units of measure of, e.g., time, concentration, kinetic parameters.

[1]The possibility to import and simulate models given in the SBML standard language is currently under development. In particular, we highlight that a well-formatted SBML file can be imported and simulated by LASSIE only if the model is entirely based on mass-action kinetics. When a SBML file is imported, the GUI automatically creates a new directory with the input files corresponding to the species and reactions described in the SBML file. To import a SBML file click File > Import SBML or press CTRL+I. Note that the python-libsml library is required to use this beta version of the SBML import module.
