# Supplementary material for: LASSIE: simulating large-scale models of biochemical systems on GPUs
Source: BMC Bioinformatics. 2017 May 10;18:246. doi: 10.1186/s12859-017-1666-0 (PMC5424297; doi:10.1186/s12859-017-1666-0)
Supplement: Supplementary file 2 — LASSIE input files and command line arguments. (PDF 221 kb) [file 12859_2017_1666_MOESM2_ESM.pdf]

ADDITIONAL FILE 2

LASSIE input files and command line arguments

A. Tangherloni, M.S. Nobile, D. Besozzi, G. Mauri, P. Cazzaniga

Input files

We provide here the complete list of input files required by LASSIE. Table 1 reports the input file name, a brief description of the file content, the format of the file, a flag indicating if the file is mandatory, the default values used by LASSIE (if applicable). In the table, N represents the number of chemical species, M represents the number of reactions, T is the number of sampling time instants in which LASSIE will save the dynamics of the system. We specify that the values stored in all files are tab-separated and all indexes are 0-based.

Table 1: Complete list of LASSIE input files

File name	*Content*	*Format*	*Optional*	*Default*
`alphabet`	Vector of chemical species names (used for plots)	N columns	Yes	X_j, j=0, ..., N-1
`left_side`	Stoichiometric matrix of the reactants[1]	M rows, N columns	No	
`right_side`	Stoichiometric matrix of the products[2]	M rows, N columns	No	
`c_vector`	Vector of kinetic constants[3]	M rows	No	
`M_0`	Vector of the initial amounts of chemical species[4]	N columns	No	
`t_vector`	Vector of sampling time instants	T rows	No	
`M_feed`	Vector of the chemical species "in feed"[5]	N columns	Yes	0
`modelkind`	Type of input model[6]	{stochastic, deterministic}	Yes	deterministic
`volume`	Reaction volume of the system[7]	1 value	Yes	
`cs_vector`	Vector of chemical species to be sampled	$K \leq N$ rows	Yes	All chemical species
`atol_vector`	Vector tolerance for Runge-Kutta-Fehlberg method	N rows	Yes	10^{-12}
`be_step`	Integration step of Backward Euler method	1 value	Yes	0.1
`newton_iter`	Maximum number of iterations allowed during each call of the Newton-Raphson method	1 value	Yes	10^4
`newton_tol`	Tolerance of Newton–Raphson method	1 value	Yes	10^{-6}
`rkf_step`	Initial integration step of Runge-Kutta-Fehlberg method	1 value	Yes	10^{-3}
`stiffness_tol`	Tolerance value to switch between Runge-Kutta-Fehlberg and Backward Euler methods	1 value	Yes	10^{-6}

[1] Left-hand side of the reactions.
[2] Right-hand side of the reactions.
[3] It can be specified both in terms of deterministic or stochastic kinetic constants associated with the reactions (see `modelkind` file).
[4] It can be specified both in terms of concentrations or molecular amounts (see `modelkind` file).
[5] This vector indicates the chemical species whose amounts must be kept constant throughout the simulation (i.e., they are assumed to be constantly fed into the system). Values in this vector are equal to 0 if the species are allowed to vary in time, they are equal to the specified amount otherwise.
[6] Deterministic: amount of chemical species given as concentration values, deterministic kinetic constants associated with the reactions; stochastic: amount of chemical species given as integer numbers of molecules, stochastic kinetic constants associated with the reactions.
[7] Required if the model is defined as stochastic in the `modelkind` file, in order to convert the stochastic kinetic constants into the deterministic formulation and the molecular amounts in concentrations.

Command line arguments

LASSIE must be launched by using the following command:
`./lassie inputFolder outputFolder listOfOptionalParameters`

- `inputFolder` is the directory which contains the input files, encoded as described above;
- `outputFolder` is the folder where the simulation output files will be saved;
- `-v` is the verbose parameter, used to obtain more information by the execution of LASSIE, e.g., the parameters currently used, the number of threads and blocks launched on the GPU, the size of the model;
- `-p value` is the parameter used to set the floating point precision, `value` can be set either to `float` or `double`. LASSIE uses double floating point precision as default.

One simple example

In this section we provide a straightforward example of LASSIE's usage, showing how to encode a very simple model following the format used by LASSIE. For the sake of clarity, we exploit here a small and simple reaction-based model to explain the functioning of LASSIE; though, we stress the fact that LASSIE is designed to simulate large-scale models based on mass-action kinetics. Let us consider a model of enzyme kinetics consisting in four molecular species (i.e., substrate S, enzyme E, intermediate complex ES and final product P) and three chemical reactions:

- $r_1 \; : \; S + E \rightarrow ES$
- $r_2 \; : \; ES \rightarrow S + E$
- $r_3 \; : \; ES \rightarrow E + P$

The stoichiometric matrices corresponding to these reactions are shown below:

Reactants (left side)

	**S**	**E**	**ES**	**P**
r_1	1	1	0	0
r_2	0	0	1	0
r_3	0	0	1	0

Products (right side)

	**S**	**E**	**ES**	**P**
r_1	0	0	1	0
r_2	1	1	0	0
r_3	0	1	0	1

In these tables, each cell contains the number of molecules of each species involved in each reaction as reactant (left side) or product (right side). Reactants and products matrices must be saved in two text files named `left_side` and `right_side`, respectively. In both files, the columns are separated with tabs and the reaction lines are separated with carriage returns, as shown in Figure 1.

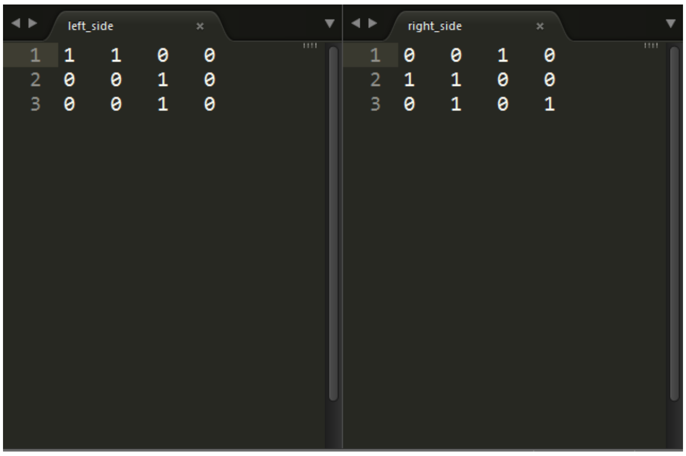

Figure 1: Example of stoichiometric matrices encoded using LASSIE's format.

LASSIE requires additional information to perform a simulation: the initial amounts of the molecular species, the kinetic constants and the sampling times. These information are provided in three different files named `M_0`, `c_vector` and `t_vector`, respectively. Please note that the order of the initial amounts in `M_0` and of the kinetic parameters in

c_vector must correspond to the order used in the stoichiometric matrices. Figure 2 shows the content of file M_0 (left), in which the initial amounts of molecular species are $S = 450$, $E = 300$, $ES = 0$, $P = 0$; the content of file c_vector (center), in which the kinetic constants are $k_1 = 0.0025$, $k_2 = 0.1$, $k_3 = 5.0$; the content of file t_vector (right), in which it is specified that LASSIE will save the dynamic behavior of the model between $t = 0$ and $t = 9.0$, saving the state of the system with intervals of 1.0 time units.

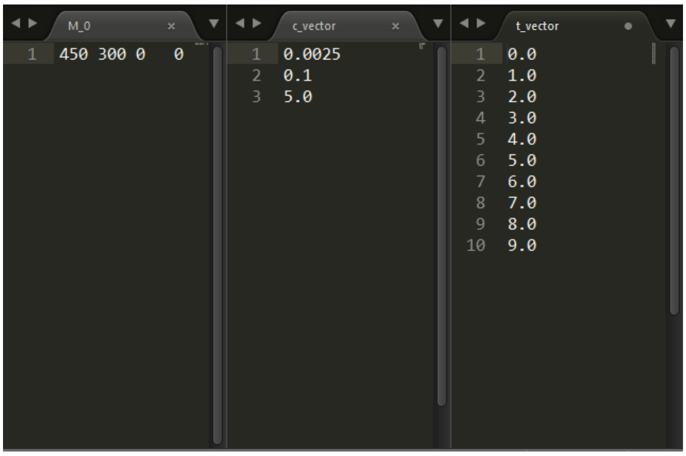

Figure 2: Example of initial amounts, kinetic parameters and sampling times files encoded using LASSIE's format.

All files described above are saved in a directory named input_model. The information contained in this directory are sufficient to perform a simulation with LASSIE, using the following command line statement:

```
./lassie input_model output_dir
```

LASSIE will parse the input files cointained in the directory and will automatically derive both the system of coupled ODEs and the Jacobian matrix corresponding to the model, assuming a mass-action kinetics. After that, the numerical integration is automatically performed, switching between explicit and implicit methods according to the stiffness of the system. When the simulation is over, the output dynamics is saved in a file named Solution within the output_dir directory.

As reported in Table 1, LASSIE accepts a variety of additional input files giving control over its internal functioning. For instance, the files atol_vector, newton_tol and stiffness_tol allow to set, respectively: the absolute tolerance for the RKF method, the tolerance used for the Newton-Raphson method, and the tolerance value used to detect the stiffness of the system.
