# Supplementary material for: LASSIE: simulating large-scale models of biochemical systems on GPUs
Source: BMC Bioinformatics. 2017 May 10;18:246. doi: 10.1186/s12859-017-1666-0 (PMC5424297; doi:10.1186/s12859-017-1666-0)
Supplement: Supplementary file 3 — Implementation of CUDA kernels for LASSIE execution workflow. (PDF 240 kb) [file 12859_2017_1666_MOESM3_ESM.pdf]

ADDITIONAL FILE 3

Implementation of CUDA kernels for LASSIE execution workflow

A. Tangherloni, M.S. Nobile, D. Besozzi, G. Mauri, P. Cazzaniga

Implementation of phase P_3

Phase P_3 implements the RKF method [5], an explicit integration algorithm with variable step-size used by each thread j to solve the j-th ODE, for $j = 0, \ldots, N-1$. This phase is implemented as 9 CUDA kernels.

During this phase, for each step two different approximated states $\mathbf{u}(t+dt)$ and $\mathbf{w}(t+dt)$ of the state $\mathbf{X}(t+dt)$ of the system are generated as follows:

$$\begin{aligned}
\mathbf{u}(t+dt) &= \mathbf{X}(t) + \frac{25}{216}\mathbf{l_1} + \frac{1408}{2565}\mathbf{l_3} + \frac{2197}{4104}\mathbf{l_4} - \frac{1}{5}\mathbf{l_5}, \\
\mathbf{w}(t+dt) &= \mathbf{X}(t) + \frac{16}{135}\mathbf{l_1} + \frac{6656}{12825}\mathbf{l_3} + \frac{28561}{56430}\mathbf{l_4} - \frac{9}{50}\mathbf{l_5} + \frac{2}{55}\mathbf{l_6},
\end{aligned} \tag{1}$$

where

$$\begin{aligned}
\mathbf{l_1} &= dtf(t, \mathbf{X}(t)), \\
\mathbf{l_2} &= dtf\left(t + \frac{1}{4}dt, \mathbf{X}(t) + \frac{1}{4}\mathbf{l_1}\right), \\
\mathbf{l_3} &= dtf\left(t + \frac{3}{8}dt, \mathbf{X}(t) + \frac{3}{32}\mathbf{l_1} + \frac{9}{32}\mathbf{l_2}\right), \\
\mathbf{l_4} &= dtf\left(t + \frac{12}{13}dt, \mathbf{X}(t) + \frac{1932}{2197}\mathbf{l_1} - \frac{7200}{2197}\mathbf{l_2} + \frac{7296}{2197}\mathbf{l_3}\right), \\
\mathbf{l_5} &= dtf\left(t + dt, \mathbf{X}(t) + \frac{439}{216}\mathbf{l_1} - 8\mathbf{l_2} + \frac{3680}{513}\mathbf{l_3} - \frac{845}{4104}\mathbf{l_4}\right), \\
\mathbf{l_6} &= dtf\left(t + \frac{1}{2}dt, \mathbf{X}(t) - \frac{8}{27}\mathbf{l_1} + 2\mathbf{l_2} - \frac{3544}{2565}\mathbf{l_3} + \frac{1859}{4104}\mathbf{l_4} - \frac{11}{40}\mathbf{l_5}\right).
\end{aligned} \tag{2}$$

To evaluate the accuracy of $\mathbf{u}$ and $\mathbf{w}$ at the current step-size dt, LASSIE exploits a user-defined vector tolerance $\boldsymbol{\varepsilon} \in \mathbf{R}^N$ (with $\varepsilon_j > 0$ for all $j = 1, \ldots, N$), and two additional arrays of type *double*, $\mathbf{ER}, \boldsymbol{\delta} \in \mathbf{R}^N$, defined as follows:

$$\mathbf{ER} = \frac{|\mathbf{w}(t+dt) - \mathbf{u}(t+dt)|}{dt}, \quad \boldsymbol{\delta} = 0.84\left(\frac{\boldsymbol{\varepsilon}}{\mathbf{ER}}\right)^{\frac{1}{4}}. \tag{3}$$

If $ER_j \leq \varepsilon_j$ for all $j = 1, \ldots, N$, then $\mathbf{u}$ is accepted as new state of the system, that is, $\mathbf{X}(t+dt) = \mathbf{u}(t+dt)$; otherwise, the solutions $\mathbf{u}$ and $\mathbf{w}$ are rejected and recalculated by using a new step-size. The new step-size is computed as $dt = dt \cdot \min\{\delta_1, \ldots, \delta_N\}$, being $\delta_1, \ldots, \delta_N$ the components of vector $\boldsymbol{\delta}$ (note that the new value of dt has to be chosen in order to satisfy the requested error tolerance for all ODEs).

Overall, phase P_3 is implemented by means of the following kernels:

- **kernel $\mathbf{K_2}$**: used to evaluate each ODE at the current state $\mathbf{X}$ of the system;
- **kernels $\mathbf{K_3}$ – $\mathbf{K_8}$**: each thread j, for $j = 0, \ldots, N-1$, computes the components $l_{1j}, \ldots, l_{6j}$ of $\mathbf{l_1}, \ldots, \mathbf{l_6}$, by invoking **kernel $\mathbf{K_2}$**;

- **kernel $\mathbf{K_9}$**: each thread j, for $j = 0, \ldots, N-1$, computes the components w_j and u_j of the approximated states $\mathbf{u}$ and $\mathbf{w}$, respectively;
- **kernel $\mathbf{K_{10}}$**: each thread j, for $j = 0, \ldots, N-1$, calculates the components ER_j and δ_j of $\mathbf{ER}$ and $\boldsymbol{\delta}$, respectively.

Implementation of phase P_5

Phase P_5 implements the BDF methods, the most widely used implicit multi-step numerical integration algorithms [8]. The general formula for a BDF can be written as

$$\sum_{i=0}^{q} \alpha_i \mathbf{X}(t - t_i) = dt\beta_0 f(t, \mathbf{X}(t)), \tag{4}$$

where the coefficients α_i (with $\alpha_0 = 1$) and β_0 are chosen according to the order q of BDF [8], and dt is user-defined. Note that, for $q > 6$, the absolute stability region of the resulting BDF methods is too small, so that such BDFs are characterized by numerical instability [3]. Therefore, BDFs with an order q greater than 6 are not used. Since each BDF is an implicit method, at each time step it requires the solution of a nonlinear system of equations, which can be solved by using the iterative Newton–Raphson method [2]. This system can be written as follows:

$$g(\mathbf{X}(t)) \equiv \mathbf{X}(t) - dt\beta_0 f(t, \mathbf{X}(t)) + c^{\mathbf{X}}(t) = 0, \tag{5}$$

where $c^{\mathbf{X}}(t) = \sum_{i=1}^{q} \alpha_i \mathbf{X}(t - t_i)$ is a constant quantity depending on previous values of the state of the system $\mathbf{X}(t)$ and on the order q.

The Newton–Raphson method allows to find successively better approximations z of the zeros of a real-valued function $f(z) = 0$, and it is repeated until a sufficiently accurate value is reached. The approximation at iteration n is calculated as follows:

$$z_{n+1} = z_n - \frac{f(z_n)}{f^{'}(z_n)}, \tag{6}$$

where $f^{'}(z_n)$ denotes the derivative of $f(z_n)$.

This idea can be extended to a system of nonlinear equations, by using the Jacobian matrix $\mathbf{J}(t, \mathbf{X}(t))$ of $f(t, \mathbf{X}(t))$. Therefore, the following system should be solved:

$$\begin{cases} \mathbf{J}(\mathbf{X}^i)\mathbf{v}\mathbf{X}^i = -f(\mathbf{X}^i) \\ \mathbf{X}^{i+1} = \mathbf{X}^i + \mathbf{v}\mathbf{X}^i, \end{cases} \tag{7}$$

where $\mathbf{v}\mathbf{X}^i$ is the vector used to update $\mathbf{X}^i$, and $\mathbf{X}^{i+1}$ at time t can be written as

$$\mathbf{X}^{i+1}(t) = \mathbf{X}^i(t) - \left(\mathbf{I} - \frac{\partial f}{\partial \mathbf{X}}\right)^{-1} g(\mathbf{X}^i(t)). \tag{8}$$

Since the calculations required to invert the matrix $\left(\mathbf{I} - \frac{\partial f}{\partial \mathbf{X}}\right)$ are computationally expensive, we can derive the following linear system:

$$\begin{cases} (\boldsymbol{I} - dt\beta_0 \frac{\partial \boldsymbol{f}}{\partial \mathbf{X}}(t, \mathbf{X}^i(t))\boldsymbol{\Delta}\mathbf{v}^i = -g(t, \mathbf{X}^i(t)) \\ \mathbf{X}^{i+1}(t) = \mathbf{X}^i(t) + \boldsymbol{\Delta}\mathbf{v}^i, \end{cases} \tag{9}$$

where $\boldsymbol{\Delta}\mathbf{v}^i$ is the vector solution of the linear system (Equation 9) at iteration i, and $\mathbf{I}$ is the identity matrix. The $\boldsymbol{\Delta}\mathbf{v}^i$ vector is used to update the iteration vector $\mathbf{X}^{i+1}(t)$ required by the Newton-Raphson method.

Since the evaluation of the Jacobian matrix at each iteration is computationally expensive, LASSIE actually exploits the modified Newton–Raphson method [7]. Thus, the iteration matrix is evaluated once

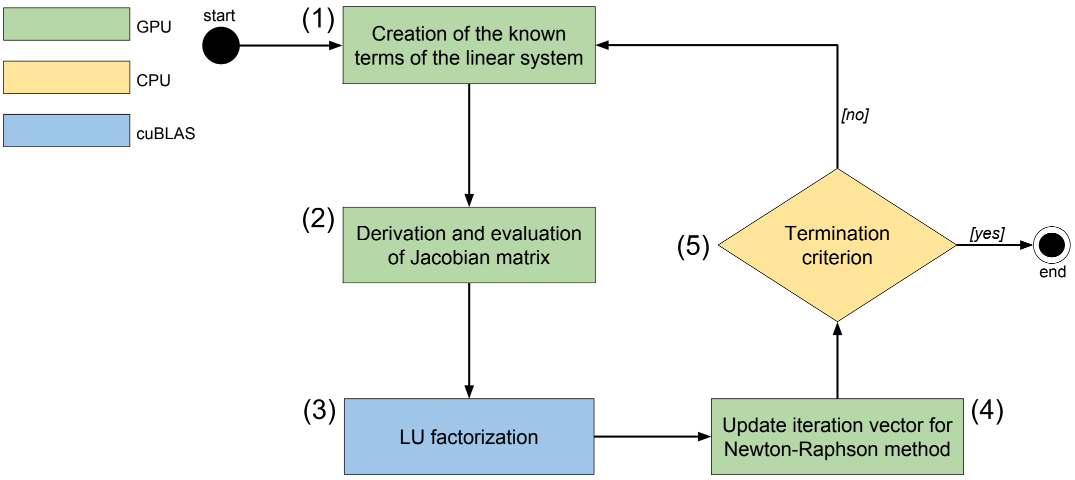

Figure 1: Flowchart of phase P_5. (1) Each thread j, for $j = 0, \ldots, N-1$, calculates the j-th known term of the linear system given in Equation 9, which is obtained from the system of non-linear equations given in Equation 7; (2) each thread j derives the j-th row of the Jacobian matrix and evaluates it on the current state of the system $\mathbf{X}$; (3) the linear system is solved by exploiting the LU factorization method supplied by cuBLAS library; (4) the solution of the linear system is used to update the iteration vector for the execution of the Newton-Raphson method; (5) steps 1 to 4 are iterated until the termination criterion is satisfied, that is, a maximum number of iterations is reached, or a value smaller than a fixed tolerance value ε_{NR} is achieved.

at the beginning of each step, based on the predicted value $\mathbf{X}^0(t)$, and it is used for all the iterations during the current step. The linear system is solved by using the LU factorization method [1], which is a direct method supplied by the Nvidia CUDA Basic Linear Algebra Subroutines (cuBLAS) [6], a GPU-accelerated version of the standard BLAS library [4]. The Newton-Raphson method is iterated until the maximum number of iterations is reached, or a sufficiently accurate value is achieved (i.e., smaller than a user-defined tolerance value ε_{NR}). When this method ends, the state of the system is updated as $\mathbf{X}(t + dt) = \mathbf{X}^{i+1}(t)$. The workflow of phase P_5 is schematized in Figure 1.

Overall, phase P_5 is implemented by means of the following kernels:

- **kernel $\mathbf{K_{11}}$**: each thread j, for $j = 0, \ldots, N-1$, derives the j-th row of the Jacobian matrix and evaluates it on the current state of the system $\mathbf{X}$;
- **kernel $\mathbf{K_{12}}$**: the Jacobian matrix is transposed in order to exploit the LU factorization method;
- **kernels $\mathbf{K_{13}} - \mathbf{K_{18}}$**: based on the order q of the BDF, LASSIE invokes one of these kernels (i.e., **kernel $\mathbf{K_{13}}$** for $q = 1$, **kernel $\mathbf{K_{14}}$** for $q = 2$, ..., **kernel $\mathbf{K_{18}}$** for $q = 6$) to calculate the known terms of the linear system;
- **kernels $\mathbf{K_{19}} - \mathbf{K_{24}}$**: each **kernel $\mathbf{K_{(18+q)}}$**, $q = 1, \ldots, 6$, performs the calculations of the q-th order BDF;
- **kernel $\mathbf{K_{25}}$**: it updates the iteration vector needed to execute the Newton-Raphson method.

References

[1] R. H. Bartels and G. H. Golub. The simplex method of linear programming using LU decomposition. *Commun. ACM*, 12(5):266–268, 1969.

[2] A. Ben-Israel. A Newton-Raphson method for the solution of systems of equations. *J. Math. Anal. Appl.*, 15(2):243–252, 1966.

[3] C. W. Gear. The control of parameters in the automatic integration of ordinary differential equations. *Int. Rep.*, 757, 1968.

[4] C. L. Lawson, R. J. Hanson, D. R. Kincaid, and F. T. Krogh. Basic linear algebra subprograms for Fortran usage. *ACM TOMS*, 5(3):308–323, 1979.

[5] J. H. Mathews and K. D. Fink. *Numerical methods using MATLAB*. Prentice-Hall Inc., Upper Saddle River, New Jersey, USA, 2004.

[6] Nvidia. cuBLAS library 7.5, May 2015.

[7] M. D. Smooke. Error estimate for the modified Newton method with applications to the solution of nonlinear, two-point boundary-value problems. *J. Optim. Theory Appl.*, 39(4):489–511, 1983.

[8] S. Thohura and A. Rahman. Numerical approach for solving stiff differential equations: A comparative study. *J. Sci. Front. Res. Math. Decision Sci.*, 13:7–18, 2013.
