# Supplementary material for: LASSIE: simulating large-scale models of biochemical systems on GPUs
Source: BMC Bioinformatics. 2017 May 10;18:246. doi: 10.1186/s12859-017-1666-0 (PMC5424297; doi:10.1186/s12859-017-1666-0)
Supplement: Supplementary file 4 — Simulation accuracy of LASSIE. (PDF 172 kb) [file 12859_2017_1666_MOESM4_ESM.pdf]

# ADDITIONAL FILE 4

# Simulation accuracy of LASSIE

A. Tangherloni, M.S. Nobile, D. Besozzi, G. Mauri, P. Cazzaniga

## Comparison between the simulation outcomes obtained by LASSIE and LSODA

The simulation accuracy of the solutions provided by LASSIE with respect to LSODA was tested by comparing the dynamics of four pivotal molecular species involved in a mass-action based model of the Ras/cAMP/PKA signal transduction pathway in yeast [1]. This model is characterized by stiffness and overall consists in 34 reactions among 30 molecular species. Figure 1 shows that the dynamics of species Ras2:GTP and cAMP (top), PKA and Pde1 (bottom) perfectly overlap, proving the accuracy of LASSIE. The simulation was executed by setting the following parameters:

LASSIE:

- tolerance of RKF method $\varepsilon_j = 10^{-12}$, $j = 1, \ldots, N$;
- first–order BDF method ($q = 1$);
- BDF integration step $dt = 0.1$;
- tolerance of Newton–Raphson method $\varepsilon_{NR} = 10^{-6}$;
- maximum number of iterations allowed during each call of the Newton-Raphson method $max_{it} = 10^4$;
- initial integration step of RKF method equal to $10^{-3}$;
- tolerance value to switch between RKF and Backward Euler methods $\varepsilon_s = 10^{-6}$.

LSODA[1]:

- relative tolerance equal to $10^{-6}$;
- absolute tolerance equal to $10^{-12}$;
- maximum number of internal steps equal to $10^4$.

## Analysis of the parameters of LASSIE

To assess the robustness of the parameterization of LASSIE (e.g., tolerance values set in all results presented in this paper), we performed an additional bunch of tests by changing one parameter at a time, and measuring the effect of the corresponding perturbations on the quality of the output dynamics and on the running time. For the execution of these tests we considered a model characterized by stiffness, representing a chain of isomerizations in which a chemical species $S_i$ (where $i = 1, \ldots, N-1$) undergoes a cascade of modifications: $S_i \longrightarrow S_{i+1}$. To be more precise, this model consists in 31 reactions among 32 chemical species, where the kinetic constant of each reaction is set to 0.1, and the initial concentration of species $S_1$ is equal to 1 (the initial concentration of all other species is equal to 0).

[1] The settings of LSODA were selected according to the most widely used values in literature, and exploited by the main computational tools (see, e.g., COPASI [2]).

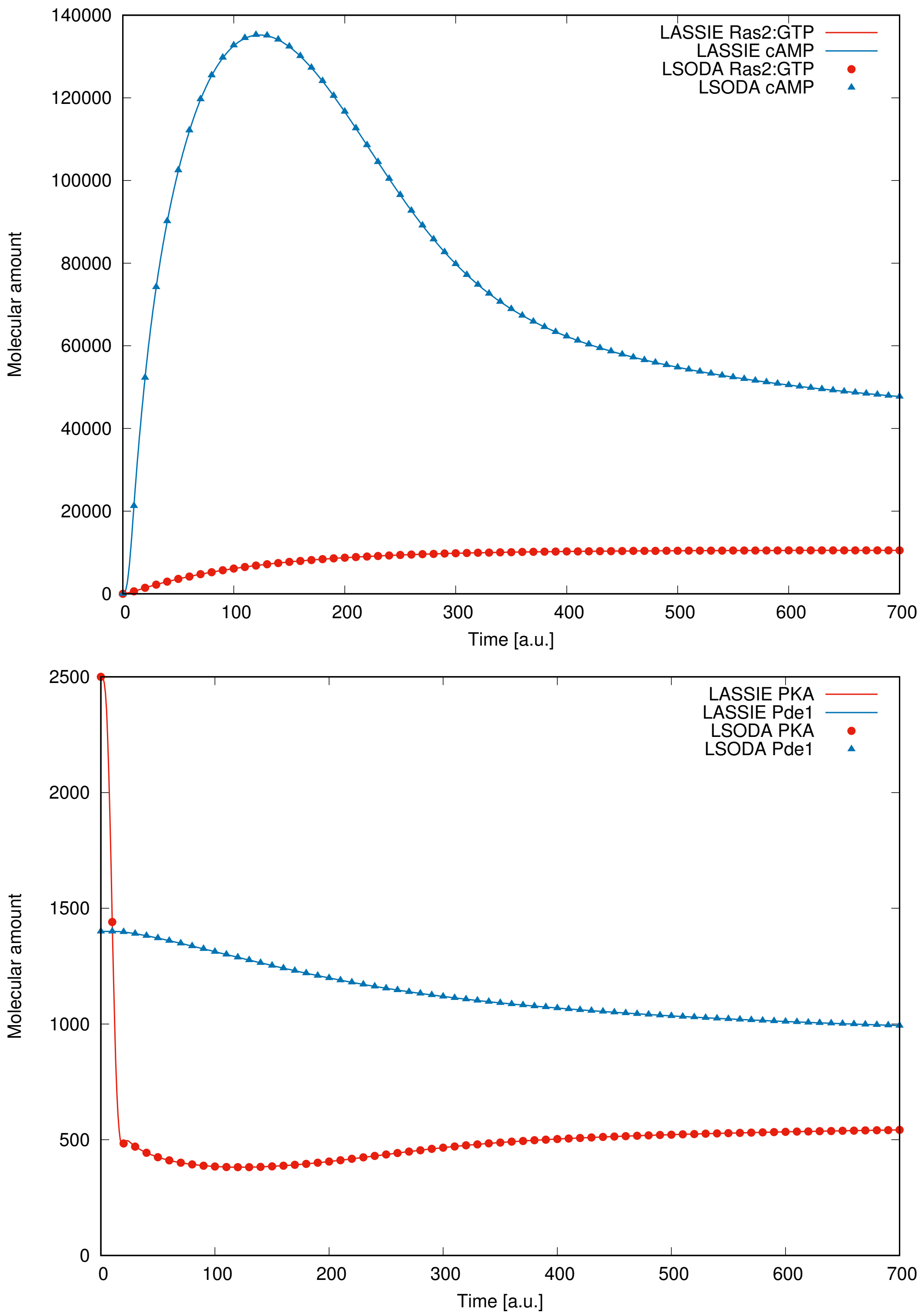


Figure 1: Comparison of the dynamics of the molecular species Ras2:GTP and cAMP (top), PKA and Pde1 (bottom) of the model of the Ras/cAMP/PKA signaling pathway in yeast, obtained by running LASSIE (solid lines) and LSODA (dots).

The variation of the tolerance value of the Newton–Raphson method, as well as the tolerance value used to switch between RKF and Backward Euler methods, have no relevant effect on the quality of the

output dynamics and on the running time of LASSIE (data not shown).

On the contrary, the variation of the tolerance value of RKF method, which is generally set as default to $\varepsilon_j = 10^{-12}$, can have a substantial effect on the output dynamics of LASSIE, as shown in Figure 2. Here, taking into account the chain of isomerizations model, the dynamics is correctly reproduced in the case of $\varepsilon_j = 10^{-12}$ and $\varepsilon_j = 10^{-18}$, while in the case of $\varepsilon_j = 10^{-6}$, the dynamics does not overlap the simulation outcome obtained by running LSODA. In addition, as the value of $\varepsilon_j$ decreases, the running time of LASSIE increases, as shown in Figure 3. However, considering that a value of $\varepsilon_j = 10^{-12}$ is sufficient to ensure a correct replication of the model dynamics, it is unnecessary to further reduce this tolerance value.

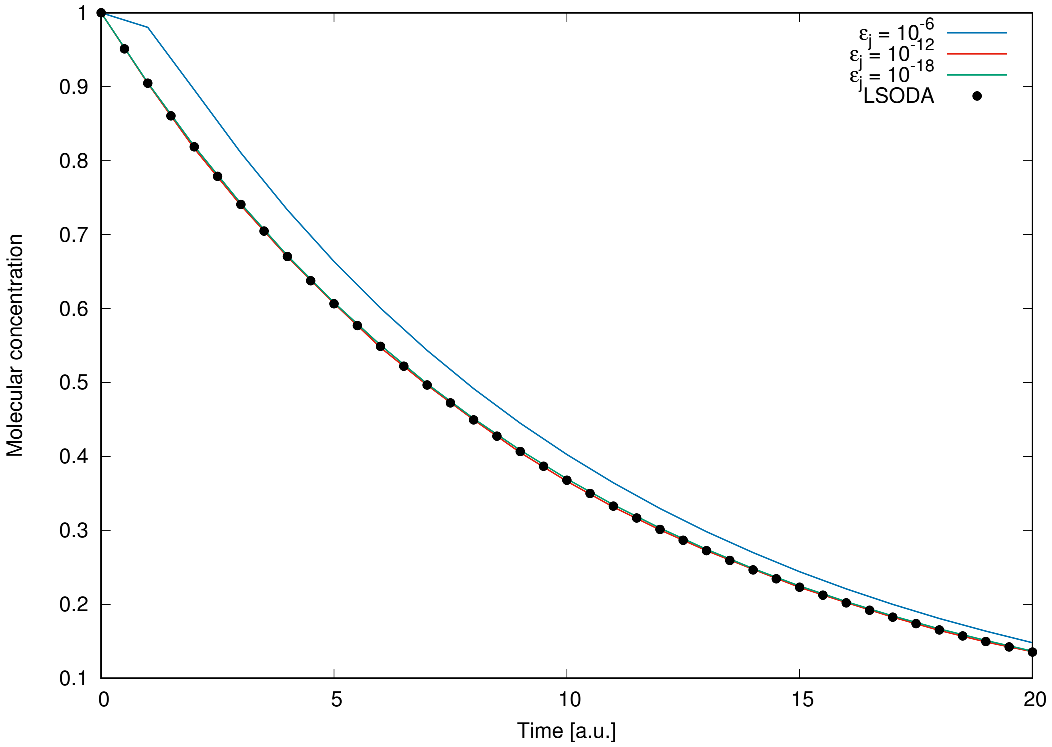


Figure 2: Comparison of the dynamics of species $S_1$ of the chain of isomerizations model obtained with LSODA (dots) and using different values of the tolerance $\varepsilon_j$ of RKF method exploited by LASSIE (solid lines).

# References


[1] P. Cazzaniga, D. Pescini, D. Besozzi, G. Mauri, S. Colombo, and E. Martegani. Modeling and stochastic simulation of the Ras/cAMP/PKA pathway in the yeast *Saccharomyces cerevisiae* evidences a key regulatory function for intracellular guanine nucleotides pools. *J. Biotechnol.*, 133(3):377–385, 2008.

[2] S. Hoops, S. Sahle, R. Gauges, C. Lee, J. Pahle, N. Simus, M. Singhal, L. Xu, P. Mendes, and U. Kummer. COPASI - a COmplex PAthway SImulator. *Bioinformatics*, 22(24):3067–3074, 2006.

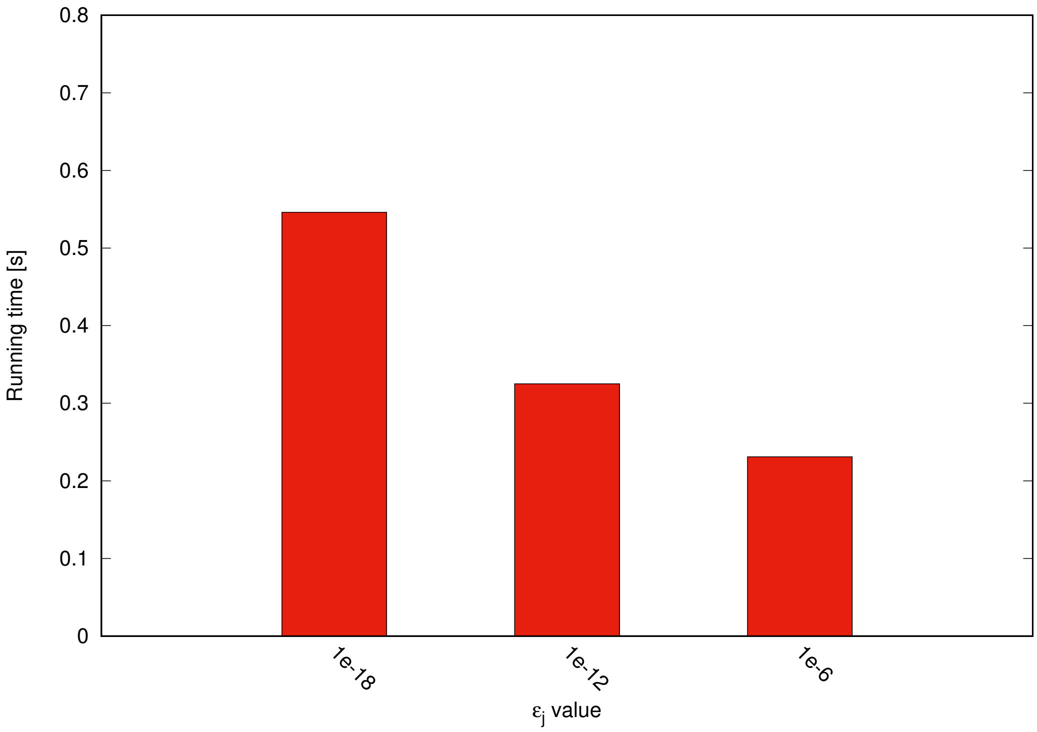


Figure 3: Running time required by LASSIE for the simulation of the chain of isomerizations model using different values of the RKF method tolerance $\varepsilon_j$.
